# Nucleosome density shapes kilobase-scale regulation by a mammalian chromatin remodeler

**DOI:** 10.1038/s41594-023-01093-6

**Published:** 2023-09-11

**Authors:** Nour J. Abdulhay, Laura J. Hsieh, Colin P. McNally, Megan S. Ostrowski, Camille M. Moore, Mythili Ketavarapu, Sivakanthan Kasinathan, Arjun S. Nanda, Ke Wu, Un Seng Chio, Ziling Zhou, Hani Goodarzi, Geeta J. Narlikar, Vijay Ramani

**Affiliations:** 1 Gladstone Institute for Data Science and Biotechnology, J. David Gladstone Institutes, San Francisco, CA USA; 2 Department of Biochemistry and Biophysics, University of California San Francisco, San Francisco, CA USA; 3 Biomedical Sciences Graduate Program, University of California San Francisco, San Francisco, CA USA; 4 Tetrad Graduate Program, University of California San Francisco, San Francisco, CA USA; 5 University of California Santa Barbara, Santa Barbara, CA USA; 6 Department of Pediatrics, Lucille Packard Children’s Hospital, Stanford University, Palo Alto, CA USA; 7 Bakar Computational Health Sciences Institute, San Francisco, CA USA

**Keywords:** Chromatin analysis, DNA, Nucleosomes, Chromatin, Chromatin remodelling

## Abstract

Nearly all essential nuclear processes act on DNA packaged into arrays of nucleosomes. However, our understanding of how these processes (for example, DNA replication, RNA transcription, chromatin extrusion and nucleosome remodeling) occur on individual chromatin arrays remains unresolved. Here, to address this deficit, we present SAMOSA-ChAAT: a massively multiplex single-molecule footprinting approach to map the primary structure of individual, reconstituted chromatin templates subject to virtually any chromatin-associated reaction. We apply this method to distinguish between competing models for chromatin remodeling by the essential imitation switch (ISWI) ATPase SNF2h: nucleosome-density-dependent spacing versus fixed-linker-length nucleosome clamping. First, we perform in vivo single-molecule nucleosome footprinting in murine embryonic stem cells, to discover that ISWI-catalyzed nucleosome spacing correlates with the underlying nucleosome density of specific epigenomic domains. To establish causality, we apply SAMOSA-ChAAT to quantify the activities of ISWI ATPase SNF2h and its parent complex ACF on reconstituted nucleosomal arrays of varying nucleosome density, at single-molecule resolution. We demonstrate that ISWI remodelers operate as density-dependent, length-sensing nucleosome sliders, whose ability to program DNA accessibility is dictated by single-molecule nucleosome density. We propose that the long-observed, context-specific regulatory effects of ISWI complexes can be explained in part by the sensing of nucleosome density within epigenomic domains. More generally, our approach promises molecule-precise views of the essential processes that shape nuclear physiology.

## Abstract (web summary)

Here authors present SAMOSA-ChAAT, a method for resolving how chromatin-interacting proteins restructure individual chromatin fibers, in high throughput and at scale. They provide evidence that the imitation switch family remodeling enzymes sense nucleosome density to program internucleosomal spacing on individual molecules.

## Main

Nucleosomes regulate most DNA-based transactions essential to life. Nuclear regulatory factors, such as sequence-specific transcription factors (TFs), polymerases, DNA repair machinery, extrusive condensin and cohesin complexes, and ATP-dependent chromatin remodeling complexes (that is, ‘chromatin remodelers’), all must navigate long stretches of nucleosomes (nucleosomal arrays) to enact cell-type-specific gene regulation. However, assessing how such regulatory factors act on individual arrays has been challenging, as methods capable of resolving such interactions are fundamentally lacking. Existing biochemical approaches for studying chromatin in bulk (for example, Förster resonance energy transfer [FRET]; gel remodeling)^1^, or at single-molecule resolution (for example, single-molecule FRET^2^ and cryogenic electron microscopy^3^), provide high-resolution views of mononucleosomes, but are generally incapable of capturing the state of individual arrays. Classical footprinting-based approaches for studying chromatin interactions are powerful, but rely on bulk averaging of nucleolytic products over many templates^4–7^. Averaging such signal is problematic, as both nucleosome positions, and the average nucleosome spacing along individual arrays, can vary substantially across a population of even identical DNA templates^8^. Single-molecule chromatin footprinting approaches developed by our group^9^ and others^10–13^ present ideal solutions to many of these issues.

**Fig. 1:**
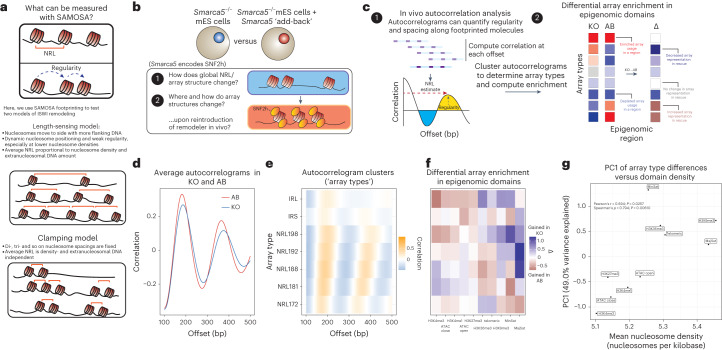
Measuring structural consequences of SNF2h rescue in mES cells at the resolution of single nucleosome arrays. **a**, Schematic overview of the structural features of nucleosome arrays measurable using the SAMOSA approach. We aimed to use SAMOSA to distinguish between two possible models of remodeling by ISWI-family, nucleosome-sliding remodelers. **b**, Experimental design of our in vivo footprinting experiment, wherein we footprint mES cells devoid of the SNF2h ISWI ATPase subunit (KO cells), and cells where the SNF2h ATPase has been reintroduced through cDNA overexpression (AB cells). We then ask how NRLs on individual fibers change across epigenomic domains. **c**, Schematic of our analytical pipeline, where we calculate single-molecule autocorrelations (left), which effectively measure the NRL and regularity of individual footprinted molecules, and then perform Leiden clustering and differential enrichment analysis (right) to determine how the reintroduction of SNF2h impacts the distribution of arrays observed across specific epigenomic domains. **d**, Average single-molecule autocorrelograms for AB (red) and KO (blue) samples. AB cells have an NRL estimate of 182 bp, and KO cells have an NRL estimate of 187 bp. **e**, Average single-molecule autocorrelograms following Leiden clustering of individual molecules. We observe seven different array types, ranging from NRL172 to NRL198, as well as two irregular array types we term IRL and IRS. **f**, Differential array enrichment across ten different epigenomic domains; red indicates gained array type usage in AB cells, and blue indicates gained array type usage in KO cells. ATAC close refers to sites that close upon rescue of SNF2h activity; ATAC open refers to sites that open upon rescue of SNF2h activity. **g**, Following PCA reduction of the matrix in **f**, we correlated PC1 against the average single-fiber nucleosome density of each domain analyzed in **f**. PC1 significantly correlates with average nucleosome density of studied domains (two-sided test).

Methodological limitations have particularly limited our understanding of ATP-dependent chromatin remodelers, such as those in the essential imitation switch (ISWI) family^14^. Mammalian ISWI complexes catalyze nucleosome sliding via the ATPase motors sucrose nonfermenting 2-homolog/-like (SNF2h/SNF2l), to facilitate DNA replication, repair, transcriptional activation and repression^15^. A key activity of ISWI complexes is to organize nucleosomes into evenly spaced arrays in the context of heterochromatin, while promoting accessibility at TF binding sites. Yet how ISWI complexes equalize spacing remains debated: some studies have proposed a ‘clamping’ model for ISWI remodeling, while others suggest a ‘length-sensing’ model of extranucleosomal DNA-dependent ISWI remodeling (Fig. 1a). The ‘clamping’ (also known as ‘ruler’) model proposes that SNF2h slides nucleosomes to create fixed internucleosomal spacing, as if via a ‘clamp.’ In this model, the HAND-SANT-SLIDE (HSS) domain of SNF2h enables clamping by binding a defined-length of linker DNA. Two key predictions of this model are that internucleosomal distances are independent of the underlying nucleosome density (that is, the average number of nucleosomes per unit length DNA) of the array, and changes in complex composition specify different clamp lengths^16–18^. In contrast, the ‘length-sensing’ model proposes that SNF2h uses the HSS to sense the length of extranucleosomal flanking DNA, and slides nucleosomes faster in the direction of longer flanking DNA. In this model, ISWI enzymes sense differences in linker lengths up to the maximal linker length bound by the HSS^1,2,19,20^. The length-sensing model predicts that steady-state internucleosomal distances generated by SNF2h will depend on pre-existing nucleosome density, and that at sufficiently low densities, SNF2h will isotropically translocate nucleosomes along template DNA. The length-sensing model also makes specific predictions of the behavior of remodeling complexes such as ACF, compared with the ATPase subunit SNF2h alone. Specifically, at lower nucleosome densities where an intact complex such as ACF is within its limit of linker-length discrimination, but the ATPase subunit is not, the remodeling complex will generate populations of evenly spaced arrays with a distribution of linker lengths. This distribution of single-array structures will be different than those generated by the ATPase alone, which will harbor fewer evenly spaced arrays, as the average internucleosomal distance on each array will be beyond the limit of linker-length discrimination. Finally, a key difference between these models is the predicted effect of remodeling on nucleosomal arrays in vivo: in the length-sensing model, nucleosome density constrains extranucleosomal DNA lengths, and thus regulates ISWI-catalyzed spacing by (1) setting spacing inversely proportional to nucleosome density, and (2) generating heterogeneous populations of both evenly and irregularly spaced arrays, particularly at low densities; in the clamping model, ISWI-catalyzed spacings should be independent of nucleosome density. Distinguishing between these models has substantial physiological relevance: first, in determining how ISWI remodelers respond to fluctuations in nucleosome density across genomic loci and during biological transitions, and second, in understanding how ISWI complexes can both repress chromatin accessibility, and enable TF binding for factors such as CCCTC-binding factor (CTCF)^21,22^.

Studies performed so far harbor unique limitations that confound resolution between the two models. Bulk and single-molecule experiments, for instance, have been performed in the context of mononucleosomes^1,19,23^, while in vitro activity measurements on arrays have relied on bulk nuclease digestion^17,18,24^. Delineation between these models is further complicated by the facts that (1) equally spaced nucleosome arrays can randomly emerge downstream of a barrier without invoking nucleosome remodeling (that is, ‘statistical’ positioning)^25^, (2) primary sequence can influence initial nucleosome positions^26^ and (3) both models will yield similar outcomes on arrays with high nucleosome density. In this Article, we leverage our single-molecule SAMOSA technology to test these models at low and high nucleosome densities, for the ISWI ATPase SNF2h and its parent complex ACF. First, we apply in vivo single-molecule chromatin footprinting to examine how genetic loss of SNF2h impacts chromatin structure in murine embryonic stem (mES) cells; we find that SNF2h loss in vivo leads to epigenomic domain-specific effects that significantly correlate with underlying average nucleosome density. Next, to test the hypothesis that nucleosome density directly impacts ISWI remodeling, we combine SAMOSA with precise biochemical reconstitution, in an approach we term SAMOSA to test Chromatin Accessibility on Assembled Templates (SAMOSA-ChAAT). Using SAMOSA-ChAAT, we perform the first array-resolved footprinting experiments demonstrating that SNF2h and ACF behave as length-sensing, nucleosome-density-dependent remodelers. Our results explain how ISWI complexes act to generate populations of evenly spaced nucleosome arrays with short, but variant, nucleosome repeat lengths (NRLs) in high-density heterochromatin; conversely, at low-density ISWI-targeted regions, remodeling slides nucleosomes to favor creation of irregular and long NRL fibers potentially supportive of TF binding. Taken as a whole, our study offers a new paradigm for single chromatin-fiber remodeling, wherein nucleosome sliding in the context of varying nucleosome density can program DNA accessibility.

## Results

### In vivo SNF2h regulation correlates with nucleosome density

**Extended Data Fig. 1:**
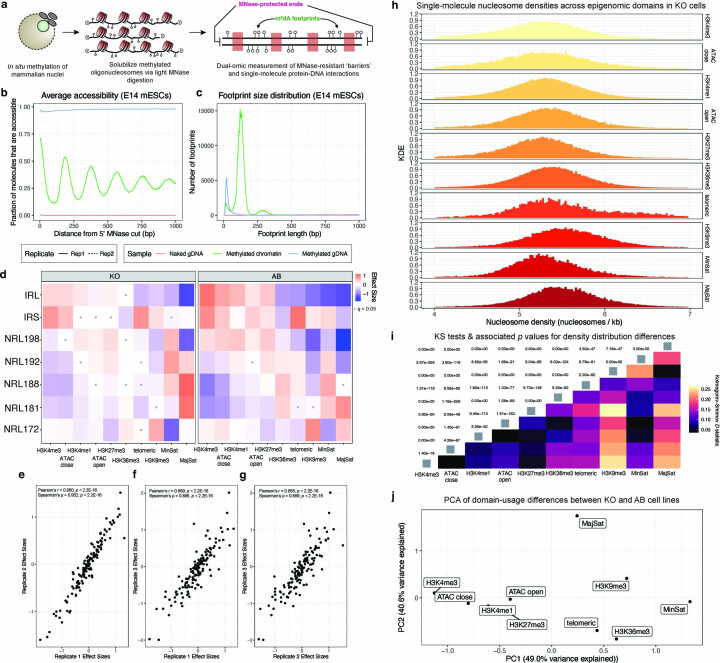
Overview of improved SAMOSA footprinting assay, fiber-type enrichment across knockout and addback cells, and measurements of single-molecule nucleosome density in knockout cells. **a)**. We improved on our previously published SAMOSA protocol by performing EcoGII methylation in intact nuclei, which we then digest with a limited MNase digestion to liberate oligonucleosomes. These molecules are then sequenced on the PacBio Sequel II platform and harbor two information types: MNase cuts that mark the position of ‘barriers’ along the genome, and m^6^dA footprints that capture protein-DNA interactions. **b)**. Our NN-HMM (Methods) can be applied to estimate chromatin accessibility on individual molecules. Shown here is data from E14 mESCs. Nucleosome periodicity is seen in footprinted chromatin, but not in positive (methylated naked DNA) and negative (unmethylated E14 gDNA) controls. The 5′ and 3′ ends of molecules are massively enriched for MNase-defined ‘barriers’ (generally, the edge of nucleosome core particles). **c)**. The NN-HMM can predict footprint sizes, which range from nucleosome length, to subnucleosomal protections indicative of transcription factor-DNA interactions. **d)**. Heat map of effect sizes for enrichment / depletion of specific fiber types across each of ten different epigenomic domains, in KO and AB mESCs. All boxes without a grey dot are significant with a *q*-value ≤ 0.05. **e.-g)**. Quantitative reproducibility of effect sizes across biological replicate KO and AB cell lines. e.), f.), and g,) show scatter plots, correlation coefficients, and *p*-values comparing paired KO / AB replicate 1 vs. replicate 2, replicate 1 vs. replicate 3, and replicate 2 vs replicate 3 effect size measurements (two-sided tests). **h)**. Distribution of single-molecule nucleosome density estimates (Methods) across each epigenomic domain studied. **i)**. Heatmap of two-sided Kolmogorov-Smirnov *D* statistics and *p*-values to test distribution differences for each epigenomic domain. All tests were statistically significant with a range of effect sizes. **j)**. Scatter plot of PC1 and PC2 resulting from PCA on the difference of the effect size matrices, shown in Fig. 1f. Points are labeled according to the represented epigenomic domain.

Substantial prior work has shown that SNF2h-containing complexes both create and repress chromatin accessibility^21,22,27,28^, but how these regulatory modes manifest on individual nucleosomal arrays in vivo remains unclear. In mammals, SNF2h (encoded by *SMARCA5/Smarca5*, hereafter referred to as SNF2h) acts as the catalytic subunit in multiple ISWI remodeling complexes^29–31^. SNF2h is dispensable in mES cells, offering a unique opportunity to study how steady-state array structure in vivo is impacted by removal and rescue of SNF2h in *trans*^22^. To build a genetic understanding of SNF2h activity at single-molecule resolution, we applied an improved version of the SAMOSA protocol and associated computational pipeline (Extended Data Fig. 1a–c and Methods) to footprint feeder-cultured mES cells devoid of SNF2h (*Smarca5*^−^^/−^ mES cells; ‘knockout’ or ‘KO’), KO cells expressing a wild-type copy of the SNF2h protein (‘rescue’ or ‘AB’)^24^, and control, feeder-free cultured E14 mES cells. Across all cell lines and including biological replicates, we sequenced 1.66 × 10^7^ individual fibers, the equivalent of ~9× haploid coverage of the mouse genome. We used these data to ask (Fig. 1b): (1) how does SNF2h loss impact the distribution of array structures genome-wide; and (2) how do SNF2h-mediated structural changes differ across the mES cell epigenome? To answer these questions, we carried out single-molecule autocorrelation analyses of footprinted molecules (Fig. 1c; left), classified single-molecule autocorrelograms into clusters using the unbiased Leiden clustering algorithm^32^, and integrated these cluster labels with ENCODE epigenomic domain definitions^33^ to calculate differential enrichment (Fig. 1c; right) across KO and rescue cell lines.

We first inspected the average single-molecule autocorrelograms of footprinted molecules from KO and rescue cells (Fig. 1d). Consistent with prior results, we found that KO cells had globally longer NRLs compared with rescue cells^22^. We then clustered single-molecule autocorrelograms to classify footprinted molecules on the bases of array regularity and NRL (Fig. 1e)^9^. Our unsupervised approach yielded seven clusters (that is, ‘array types’)—five regular array types ranging in NRL from ~172 bp to ~198 bp, and two irregular array types with weak nucleosome phasing (IRS (irregular short NRL); IRL (irregular long NRL)). We then computed enrichment of each array type across ten different epigenomic domains, almost all of which are expected to be impacted by defects in ISWI remodeling (H3K4me3 (ref. ^34^), H3K4me1, H3K36me3 (ref. ^35^), H3K27me3 (ref. ^28^), H3K9me3 (ref. ^36^), bulk differential ATAC-seq peaks^22^, telomeric sequence^36^, major satellite^36^ and minor satellite^36^; Extended Data Fig. 1d), and calculated differential enrichment between genotypes (Fig. 1e). Importantly, all observed patterns were highly quantitatively reproducible across replicate experiments (Extended Data Fig. 1e–g and Supplementary Table 1). Intriguingly, we found that the reintroduction of SNF2h in rescue cells had domain-specific effects (Fig. 1f). At predicted active promoters, for example, the addition of SNF2h leads to increased representation of ‘irregular’ and long NRL arrays; at predicted H3K36me3 regions (that is, regions where reads mapped sufficiently downstream of the promoter), SNF2h increased the representation of intermediate-length NRL arrays; finally, at typically unmappable heterochromatic major and minor satellite sequences, the addition of SNF2h led to increased representation of short NRL arrays, consistent with SNF2h condensing chromatin in this context. These results reveal that single-molecule SNF2h activity in vivo correlates on epigenome-specific features.

SNF2h depends both on nucleosomal substrate cues and myriad cofactors, all of which could impart specific activity within different epigenomic domains. We tested whether nucleosome density (that is, the average number of nucleosomes per unit length DNA), and thus, extranucleosomal DNA availability, might additionally be associated with observed changes in array type usage. SAMOSA and similar experimental workflows allow for single-molecule estimates of nucleosome density^37^: examining the average nucleosome densities of footprinted molecules falling within each region, we found that epigenomic domains differ subtly, but significantly, in average single-molecule nucleosome density (ranging in 5.10 ± 0.836 nucleosomes per kilobasepair (kbp) in H3K4me3 regions to 5.46 ± 0.805 nucleosomes kbp^−1^ in H3K9me3 regions; distributions in Extended Data Fig. 1h; Kolmogorov–Smirnov effect sizes and *P* values tabulated in Extended Data Fig. 1i). To correlate density against SNF2h effects, we performed principal components analysis (PCA) on the differential enrichment matrix (Extended Data Fig. 1j). and examined correlation between the first principal component, PC1, and mean nucleosome density in each epigenomic domain (Fig. 1g). PC1, which accounts for 49.0% of the variance in the differential enrichment matrix, strongly and significantly correlated significantly with the mean nucleosome density within each domain (Pearson’s *r* = 0.694, *P* = 0.0257; Spearman’s *ρ* = 0.794, *P* = 0.00610). Together, these analyses suggest that nucleosome density is quantitatively correlated with the regulatory output of SNF2h in vivo.

### SAMOSA footprinting of reconstituted murine nucleosome arrays

**Fig. 2:**
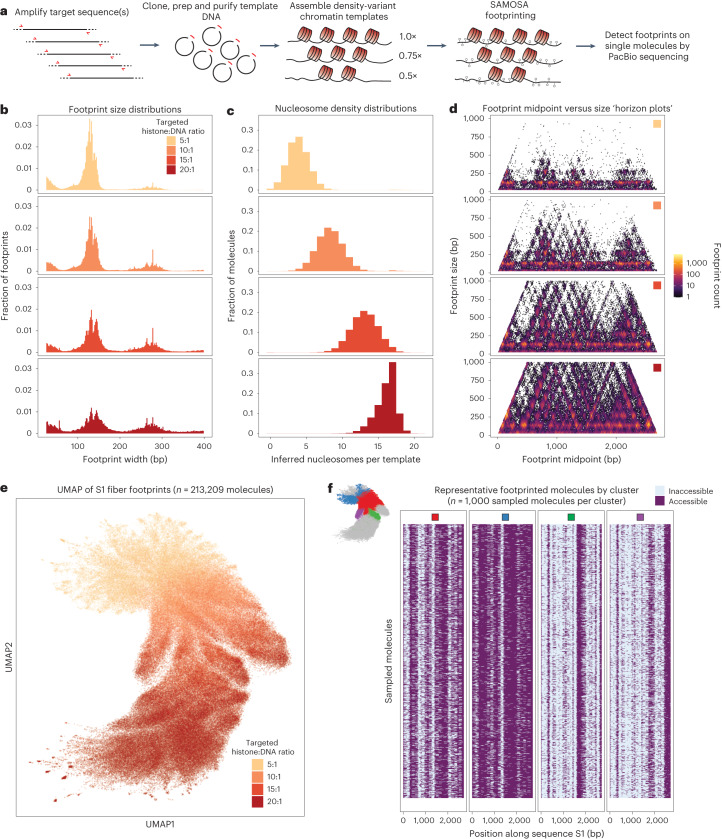
SAMOSA-ChAAT enables massively multiplex dissection of single-fiber nucleosome positioning on in vitro reconstituted genomic chromatin fibers. **a**, Schematic overview of the SAMOSA-ChAAT protocol, wherein genomic sequences are cloned, purified and assembled into chromatin fibers with desired biochemical properties (for example, nucleosome density) through SGD. Fibers are then footprinted with a nonspecific adenine methyltransferase and sequenced on the PacBio platform to assess single-molecule nucleosome positioning. **b**, A custom analytical pipeline enables detection of methyltransferase footprints on sequenced fibers. Footprint sizes from SAMOSA-ChAAT experiments carried out at varying nucleosome densities follow closely with expected nucleosome sizes, plus expected ‘breathing’ of DNA around the histone octamer, with the extent of breathing decreasing as nucleosome density increases. **c**, SAMOSA-ChAAT data enable direct estimation of the absolute number of nucleosomes per footprinted fiber, which track well with expected nucleosome densities based on targeted octamer: DNA ratios during SGD. **d**, Footprint length versus midpoint ‘horizon’ plots for footprinted fibers. Average nucleosome positions display sequence dependencies. **e**, UMAP dimensionality reduction of fiber accessibility data. UMAP patterns recapitulate known differences in nucleosome density in footprinted fibers. **f**, Visualization of a subset of sampled molecules following Leiden clustering of single molecule data. Individual Leiden clusters (cluster positions inset) capture mutually exclusive nucleosome positions consequent of chromatin fiber assembly.

The observation that nucleosome density—and by extension, availability of extranucleosomal DNA—correlates with SNF2h activity in vivo suggests that nucleosome density might directly impact nucleosomal spacing by ISWI. However, testing such a hypothesis in vivo is intractable, as (1) engineering domain-specific histone concentrations in mammalian nuclei is currently impossible, and (2) ISWI complexes interact with many sequence-specific and nonspecific *trans* regulators in a domain-specific manner. Determining the direct impact of nucleosome density on ISWI remodeling thus necessitates biochemical reconstitution. Prior biochemical studies on arrays have used repetitive DNAs containing a nucleosome positioning sequence such as Widom 601 (refs. ^38,39^), or arrays reconstituted on yeast genomic DNA^16,24,40^. We previously demonstrated that our SAMOSA protocol could accurately resolve single-fiber nucleosome footprints on 601-based chromatin arrays^9^. To enable study of more native-like chromatin, we extended the SAMOSA approach to footprint chromatin reconstituted on mammalian genomic sequences through salt gradient dialysis (SGD). We devised a general workflow we term SAMOSA-ChAAT (Fig. 2a), in which arrays with desired biochemical properties (for example, nucleosome density) are assembled from genomic templates, subjected to the SAMOSA m^6^dA footprinting protocol and sequenced on the PacBio Sequel II to natively detect m^6^dA modifications reflective of accessible DNA bases.

As proof-of-concept for this approach, we cloned two ~3 kilobase sequences from the *M. musculus* genome (hereafter, sequences ‘S1’ and ‘S2’), carried out the SAMOSA-ChAAT workflow across four specified histone octamer:DNA molar ratios and sequenced resulting molecules and controls to high depth (samples and sequencing depths summarized in Supplementary Table 2). Consistent with the assembly of histones into nucleosome core particles with varying degrees of ‘breathability,’ we were able to call stretches of unmethylated DNA on sequenced molecules (that is, ‘footprints’) ranging from ~120 to 160 nucleotides (nts) in size (Fig. 2b), in addition to short (<30 nt) footprints suggestive of nonspecific histone–DNA interactions (for example, H2A/H2B-DNA). Footprint sizes increased along with chromatin density, suggesting that higher nucleosome densities promote formation of closely spaced di- and trinucleosome structures. Our data also enable estimates of the number of nucleosomes per individual template (that is, ‘nucleosome density’); accordingly, inferred nucleosome counts on single molecules matched targeted assembly extents (Fig. 2c; values reported as mean ± standard deviation in Supplementary Table 3).

Nucleosome assembly can be influenced by the underlying shape and rigidity of template DNA, which varies strongly as a function of DNA sequence^29^. To ascertain patterns of favored nucleosome positioning in bulk, we generated footprint length versus footprint midpoint ‘horizon plots’ (analogous to fragment length versus midpoint ‘V-plots’^30^) for each assembly condition and sequence (Fig. 2d). Our approach allows for explicit mapping and classification of footprints of all sizes as a function of target sequence, clearly revealing both sequence-directed nucleosome positioning, and regions that favor formation of closely packed primary structures (for example, dinucleosomes with virtually no intervening linker DNA).

**Extended Data Fig. 2:**
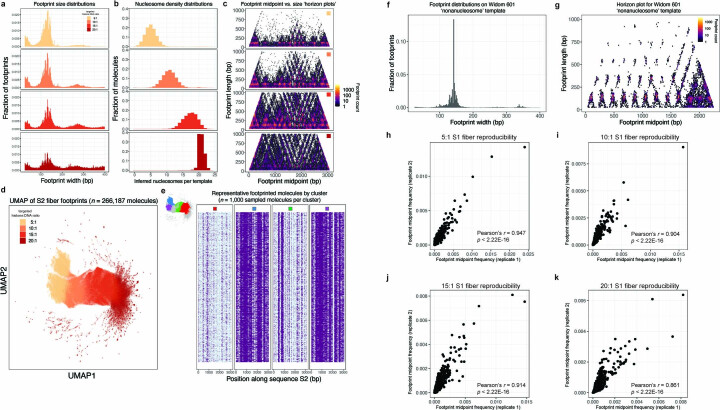
Generalizability and reproducibility of the SAMOSA-ChAAT protocol. **a)**. As in Fig. 2b, but for a completely different murine sequence (‘S2’). Footprint sizes from SAMOSA-ChAAT experiments carried out at varying nucleosome densities follow closely with expected nucleosome sizes, plus expected ‘breathing’ of DNA around the histone octamer, with the extent of breathing decreasing as nucleosome density increases. **b)**. SAMOSA-ChAAT data enables direct estimation of the absolute number of nucleosomes per footprinted S2 fiber. **c)**. Footprint length vs. midpoint ‘horizon’ plots for footprinted S2 fibers. **d)**. UMAP dimensionality reduction of S2 fiber accessibility data. **e)**. Visualization of a subset of sampled molecules following Leiden clustering of single molecule data. **f-g)**. Widom 601 nonanucleosomal fiber data from ref. ^9^ was reprocessed using the NN-HMM. Called footprints are the expected length of 601-assembled nucleosomes (**f**), and horizon plots reveal positioned nucleosomes at expected positions **(g). h–k)**. Correlation of footprint abundances for S1 fibers of each density across two replicates (different salt gradient dialysis preps).

To move beyond these bulk averages, we next explored our data at single-molecule resolution (Fig. 2e,f) using Uniform Manifold Approximation and Projection (UMAP) dimensionality reduction^41^ and Leiden community detection^32^. We found (1) that UMAP projections capture differences in assembly extent (Fig. 2e), and (2) that unbiased clustering enables detection of mutually exclusive nucleosome positions for molecules from SGD preparations (see purple and green clusters in Fig. 2f). Importantly, our data satisfy a wide set of controls. First, our footprint-size analyses, nucleosome-density measurements, horizon plot visualizations, UMAP reductions and cluster profiles were all consistent for the completely different sequence S2 (Extended Data Fig. 2a–e). Second, our analytical pipeline accurately detected expected footprint sizes and positions from Widom 601 chromatin fibers with known dyad positions (Extended Data Fig. 2f,g), albeit with a longer mononucleosome footprint size consistent with less DNA breathing on Widom 601 nucleosomes^26^. Finally, our nucleosome occupancy measurements were highly quantitatively reproducible across replicates (Extended Data Fig. 2h–k). Together, these data demonstrate the sensitivity, reproducibility and generalizability of the SAMOSA-ChAAT approach.

### Chromatin remodeling reaction outcomes at single-fiber resolution

We next used SAMOSA-ChAAT to study ATP-dependent chromatin remodeling at single chromatin fiber resolution. Across several multiplexed sequencing runs, we surveyed the core SNF2h ATPase alone, and the heterodimeric ACF complex (composed of SNF2h and ACF1), using two different stoichiometries with respect to mononucleosomes on arrays, at two different timepoints (15 min and 75 min, which represent >3 and >15 half-times, respectively). As controls, we also footprinted SNF2h in a ‘pre-catalytic’ state on arrays (that is, SNF2h(−)ATP), an uncatalyzed state where ADP was added instead of ATP (SNF2h(+)ADP), and predicted ‘multiple-turnover’ conditions where [SNF2h] < [mononucleosome]. To demonstrate reproducibility, we also performed a subset of our SNF2h-remodeling experiments on S2 arrays. Including all replicate and control experiments, and after filtering out molecules that failed quality control, we analyzed 3.25 × 10^6^ footprinted molecules, amounting to a single-molecule fold coverage of 1.80 × 10^6^-fold and 1.45 × 10^6^-fold for templates S1 and S2, respectively (Supplementary Table 2).

**Fig. 3:**
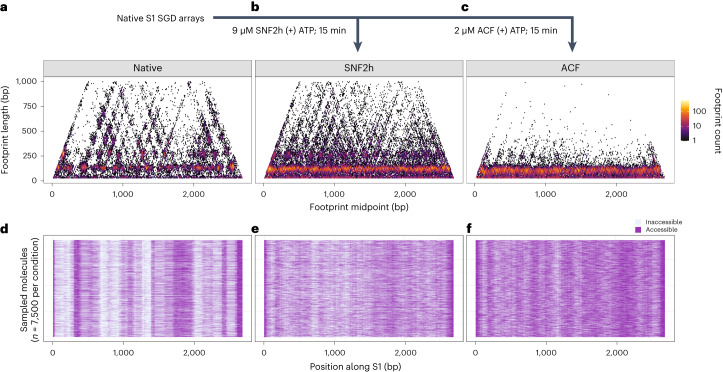
SAMOSA-ChAAT reveals chromatin remodeling outcomes at single-fiber resolution. **a**–**c**, Footprint length versus footprint midpoint horizon plots comparing native S1 fibers with between 5 and 16 nucleosomes per template (**a**), S1 fibers remodeled with 9 µM SNF2h for 15 min (**b**) and S1 fibers remodeled with 2 µM ACF for 15 min (**c**). **d**–**f**, Sampled single-molecule data, with the same experimental conditions as above (with **d**, **e** and **f** corresponding to the conditions in **a**, **b** and **c**, respectively).

**Extended Data Fig. 3:**
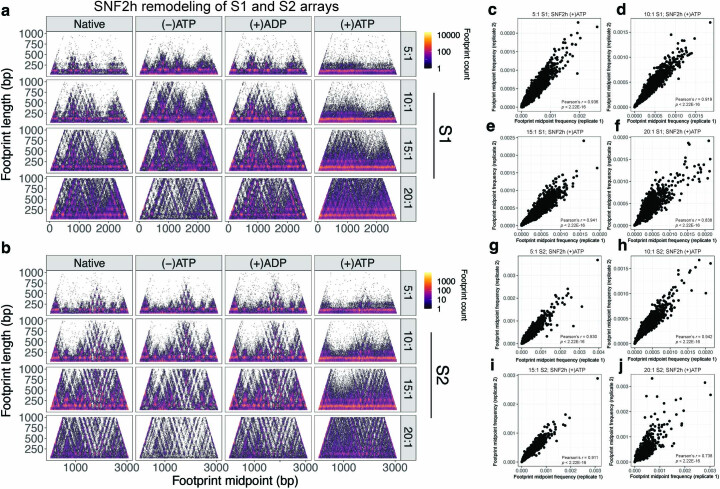
Reproducibility of SAMOSA-ChAAT remodeling experiments and horizon plots for all catalytic conditions tested. **a-b**). Horizon plots for S1 (**a**) and S2 (**b**) fibers, for native, pre-catalytic, (+)ADP, and remodeled fibers (all averages are over single-turnover experiments; multi-turnover data is omitted for this visualization). **c–j)**. Scatter plots and associated Pearson’s *r* values for correlations between two biological replicate remodeling experiments, for each density tested, for both S1 (**c-f**) and S2 (**g-j**) arrays.

We focused on exploring SNF2h and ACF remodeling of S1 fibers between 5 and 16 nucleosomes per template (1.78 nucleosomes kbp^−1^ to 5.91 nucleosomes kbp^−1^). This captures the range of densities we observe in vivo. To examine whether our assay could capture the impacts of remodeling, we visualized the bulk consequences of remodeling fibers through horizon plots (Fig. 3a–c). We found that SNF2h remodeling decreases sequence-dependent nucleosome positioning on fibers—nucleosome-sized footprint midpoints occupied virtually all possible positions along the sequences, overriding observed sequence dependencies on native fibers consistent with studies on mononucleosomes^42^. Next, we performed visual inspection of individual sampled fibers before and after remodeling (Fig. 3d–f). We observed that remodeling qualitatively increased spacing between nucleosomes on sampled fibers, and also observed the formation of what appear to be evenly spaced nucleosomal arrays in ACF remodeled samples (Fig. 3f). Importantly, several aspects of our remodeling data recapitulate existing knowledge of how ISWI binds and remodels mononucleosomes: for instance, remodeling did not substantially impact the estimated numbers of nucleosomes per template, consistent with ISWI remodelers predominantly sliding, not evicting or loading nucleosomes (Supplementary Table 3), and the precatalytic condition (SNF2h(−)ATP) yielded slightly larger footprints on average but little change in preferred nucleosome positions on templates, consistent with the HSS domain of SNF2h interrogating DNA flanking the nucleosome^20,43,44^ (Extended Data Fig. 3a). Finally, our SNF2h remodeling results were qualitatively reproducible on the completely different S2 sequence (Extended Data Fig. 3b), and highly quantitatively reproducible across biological replicates (Extended Data Fig. 3c–j). Together, these experiments demonstrate that SAMOSA-ChAAT can reproducibly quantify the outcomes of chromatin remodeling reactions at single-array resolution.

### SNF2h and ACF catalyze density-dependent array spacing

We next used our data to distinguish between ‘clamping’ versus ‘length-sensing’ models of ISWI remodeling (Fig. 1a). Intuitively, ‘clamping’ should evenly space adjacent nucleosomes at a fixed ‘ruler’ length such that average NRL is independent of array density; remodeling via ‘length-sensing,’ conversely, should space nucleosomes across individual fibers with average NRLs inversely proportional to array density, and the abundance of regularly spaced fibers in a population should vary depending on the ‘length-sensing’ distance. We reasoned that these patterns would be evident in single-molecule autocorrelograms derived from SAMOSA-ChAAT data, as the position of an autocorrelogram peak either on the average signal from all single-molecule autocorrelograms from molecules of equal density (‘per-density’ peak positions), or the autocorrelogram peak of each individual single-molecule autocorrelogram (‘per-molecule’ peak positions) should provide reasonable estimates of the average distance between nucleosomes on per-density/per-molecule bases, respectively.

**Fig. 4:**
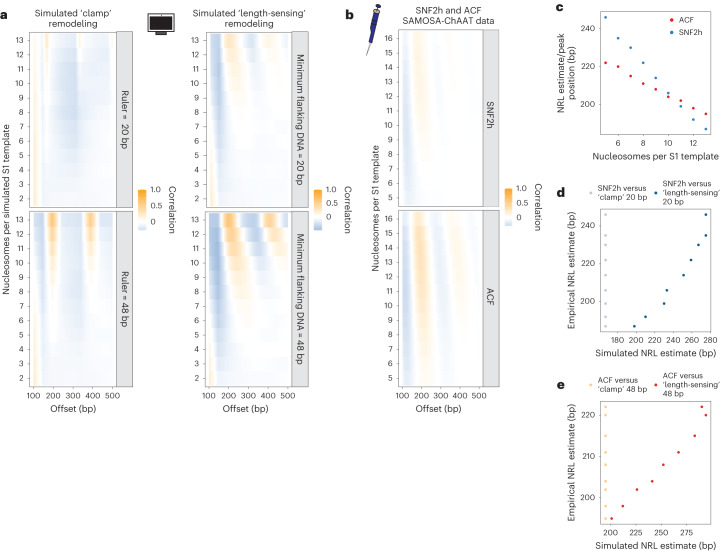
An integrative approach to test the density dependence of ISWI remodeling. **a**, We employed a Monte-Carlo simulation to simulate S1-length nucleosomal arrays with 2–13 randomly positioned nucleosomes, and then subjected these fibers to in silico ‘clamp’ remodeling (left) or ‘length-sensing’ remodeling (right), at two different ruler/flanking length cutoffs: 20 bp (top) or 48 bp (bottom). We then plotted the single-molecule autocorrelograms of simulated, remodeled molecules and plotted the average autocorrelogram for each simulated density (*y* axis) as a function of offset (*x* axis). **b**, Single-molecule autocorrelograms for empirical data for SNF2h (top) and ACF (bottom). Data from 9 µM SNF2h remodeling and all collected ACF data shown here can be used to estimate relative spacing and regularity of single, footprinted chromatin fibers. **c**, Mean NRL estimate for arrays with 5–13 nucleosomes per template, as a function of density for SNF2h (blue) and ACF (red). **d**, Mean NRL estimates for arrays with 5–13 nucleosomes per template as a function of simulated NRL estimates (20 bp simulations) for SNF2h-remodeled templates. Length-sensing correlation shown in dark blue, and clamp correlation shown in light blue. **e**, Mean NRL estimates for arrays with 5–13 nucleosomes per template as a function of simulated NRL estimates (48 bp simulations) for ACF-remodeled templates. Length-sensing correlation shown in dark red, and clamp correlation shown in light red. Source data

**Extended Data Fig. 4:**
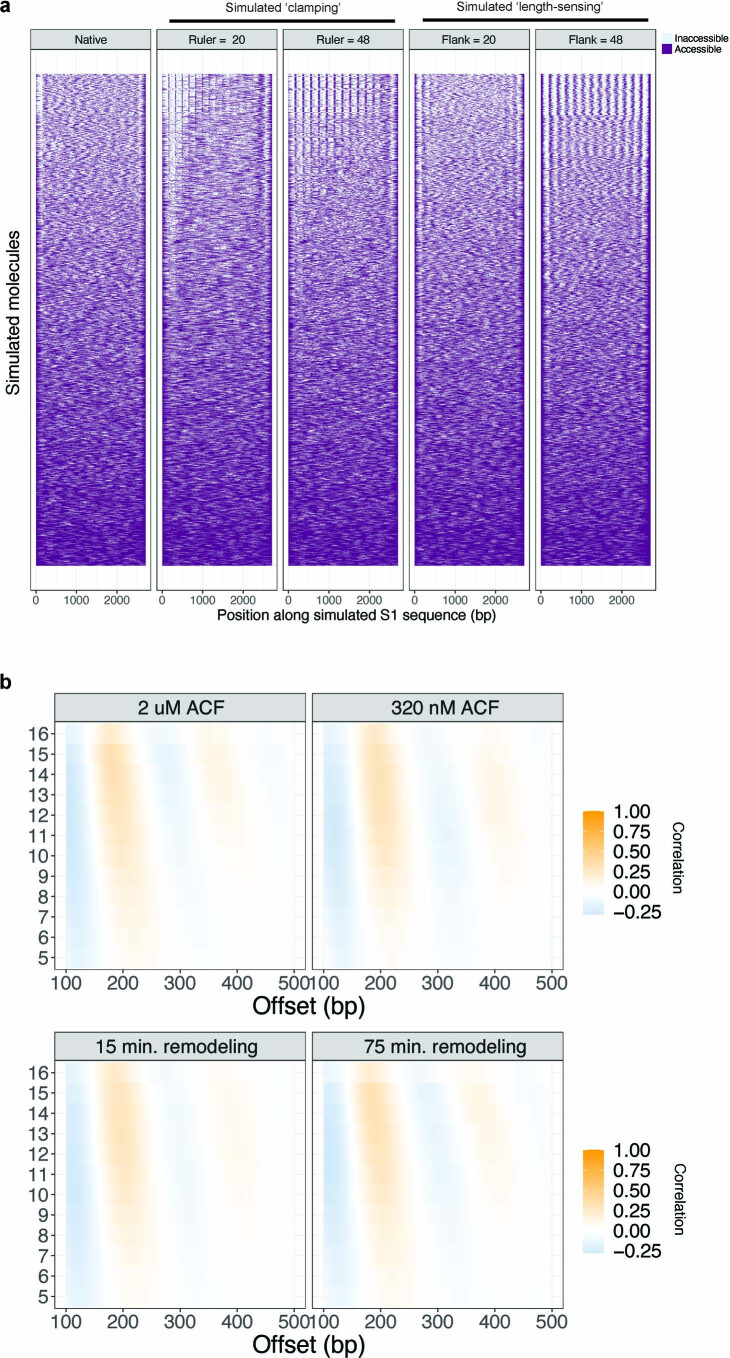
Discerning between models of ISWI remodeling through simulation and additional experimental conditions. **a)**. Heatmap representation of simulated S1 nucleosomal arrays generated through a Monte-Carlo simulation. Column 1 represents simulated ‘native’ arrays; Column 2 represents simulated ‘clamp’ remodeled arrays with a ruler length of 20 bp; Column 3 represents simulated ‘clamp’ remodeled arrays with a ruler length of 48 bp; Column 4 represents simulated ‘length-sensing’ remodeled arrays with a minimum flanking DNA length of 20 bp; Column 5 represents simulated ‘length-sensing’ remodeled arrays with a minimum flanking DNA length of 48 bp. **b)**. The observation of density-dependent NRL scaling in ACF-remodeled products is neither impacted by ACF: mononucleosome stoichiometry (**top**) nor remodeling time (**bottom**). Source data

To confirm the sensitivity of this approach, we implemented a simple Monte-Carlo simulation to first predict how remodeled arrays generated by each process may look. We simulated variable density arrays on S1-length DNA templates, and subjected these arrays to remodeling by one of two distinct processes in silico: ‘clamping,’ wherein nucleosomes falling within a specified distance are spaced against the 5′ most nucleosome (that is, ‘barrier’) at a ‘ruler’ distance, or ‘length-sensing’, wherein nucleosomes are iteratively translocated in a direction dependent on availability of extranucleosomal DNA. While these simulations are not expected to recapitulate all predicted aspects of either remodeling process (for assumptions, see Methods), they provide an easily interpretable framework for both visualizing possible patterns of array remodeling. In total, we simulated 3,000 fibers ranging in density from 2 to 14 nucleosomes. We then remodeled these arrays in silico using two different ‘ruler’ or ‘length-sensing’ distances (20 nt and 48 nt, bounded by estimates from ref. ^1^; Extended Data Fig. 4), performed ‘single-molecule’ autocorrelation analysis and examined resulting ‘per-density’ autocorrelogram averages (Fig. 4a). As expected, the two processes generate different patterns: if SNF2h and/or ACF act as a clamp, per-density autocorrelograms demonstrate a peak at a single fixed distance (Fig. 4a; left), even at low densities. Conversely, if SNF2h and/or ACF act via a length-sensing mechanism, average autocorrelogram peak values vary inversely with nucleosome density, and evenly spaced arrays are formed at lower nucleosome densities with longer length-sensing distances (Fig. 4a; right). These simulations confirm that ‘per-density’ single-molecule autocorrelation analysis of SAMOSA-ChAAT data can definitively distinguish between these two models.

**Extended Data Fig. 5:**
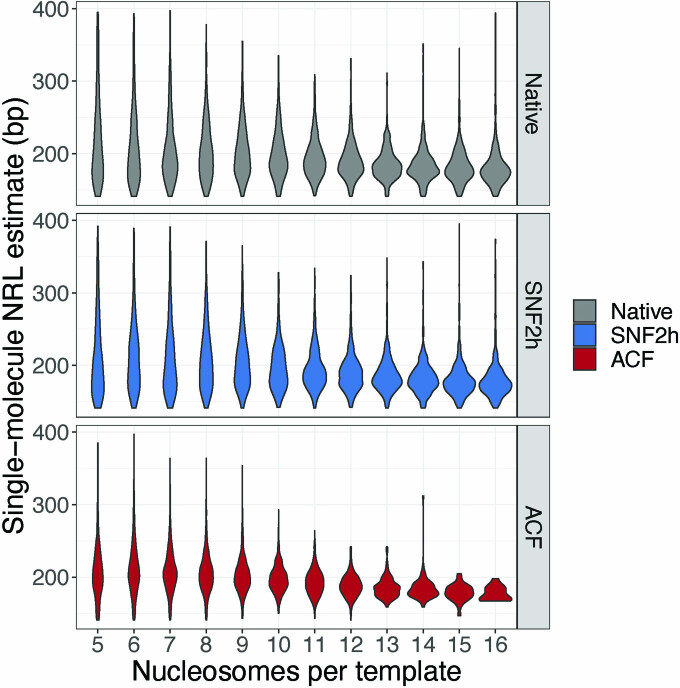
Violin plots of per-density single-molecule NRL estimates for native (un-remodeled), SNF2h-remodeled, and ACF remodeled S1 arrays. Means and standard deviation for all distributions shown in Supplementary Table 4.

We next computed single-molecule autocorrelations for SNF2h- and ACF-remodeled S1 fibers and similarly visualized ‘per-density’ autocorrelograms (Fig. 4b, Extended Data Fig. 5 and Supplementary Table 4; single-molecule nucleosome density calculated as for native S1 fibers). Our data for SNF2h and ACF strongly support the ‘length-sensing’ model: first, autocorrelation peak positions for the average autocorrelograms vary inversely with nucleosome density (Fig. 4c; SNF2h: Pearson’s *r* = −1.00, *P* = 1.27 × 10^−9^; ACF: Pearson’s *r* = −0.997, *P* = 3.29 × 10^−9^), and second, SNF2h (whose sensitivity to extranucleosomal DNA extends only to ~25 bp; ref. ^1^) demonstrates higher variance in ‘per-molecule’ peak positions (Extended Data Fig. 5) at lower nucleosome densities (for example, 9–12 nucleosomes per template) compared with ACF-remodeled products.

Reassuringly, our data are also concordant with aspects of our simulation of ‘length-sensing’, and are not at all concordant with elements of ‘clamping’. The ‘per-density’ NRL from our empirical data correlated with our ‘length-sensing’ simulation at both tested spacing parameters (Fig. 4d,e; dark color) and did not correlate with peak estimates from ‘clamping’ simulations (Fig. 4d,e; light color). Our results do, however, demonstrate how both models generate similar spacings at high densities, but differ substantially at lower densities (for example, see similar peak positions for each model in Fig. 4d,e). Importantly, density dependence of ISWI-catalyzed spacing in our experiments could also be shown by an analysis independent of autocorrelation: measurement of dinucleosomal spacings on individual arrays, which we visualized as movies where individual frames represent dinucleosomal spacings from arrays with a particular nucleosome density (Supplementary Videos 1–3). Finally, neither remodeling time nor remodeler:nucleosome stoichiometry impacted our observation of density-dependent ACF remodeling (Extended Data Fig. 4b). Together, these data are consistent with ISWI translocating nucleosomes towards longer linker DNA, in accordance with a density-dependent, length-sensing mechanism of action.

### Heterogeneous outcomes of density-dependent ISWI remodeling

**Fig. 5:**
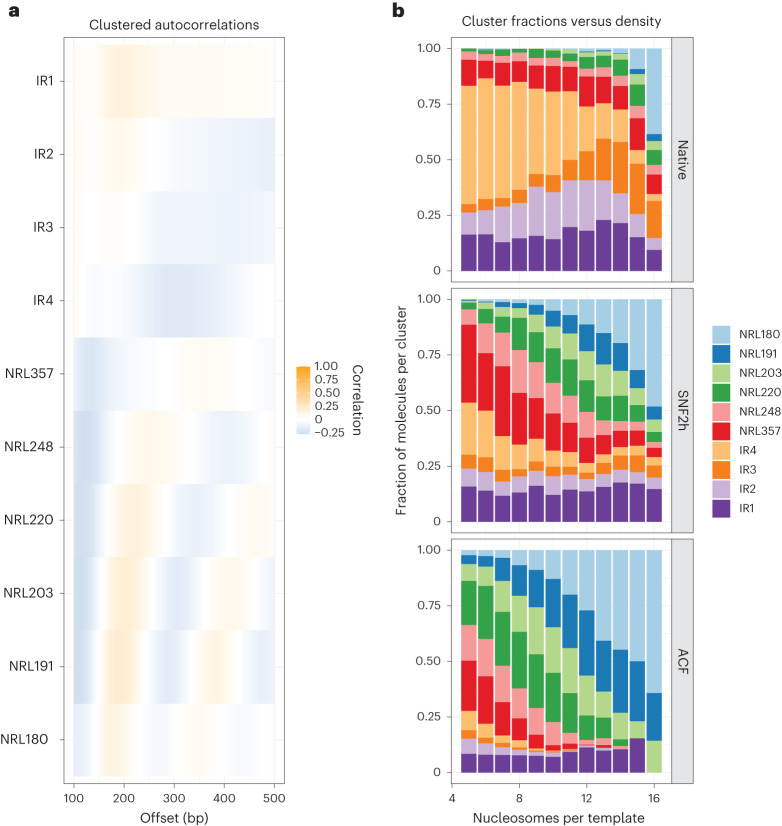
ISWI remodeling outcomes are heterogeneous, are density dependent and act on pre-existing nucleosome array structures. **a**, Clustered autocorrelograms for sampled native, SNF2h-remodeled and ACF-remodeled S1 arrays. Clusters capture arrays with NRLs ranging from 180 to 357 bp, as well as four ‘irregular’ (IR) array types with no detectable NRL/regularity. **b**, Stacked bar chart representation of cluster representation, plotted as a function of nucleosome density.

The length-sensing model predicts that ISWI enzymes will catalyze a diversity of array structures as a function of density, and that this distribution of structures will differ for the SNF2h ATPase alone compared with the intact ACF complex. To move beyond ‘per-density’ and ‘per-molecule’ peak estimates and better quantify heterogeneous remodeling outcomes, we again employed Leiden clustering of single-molecule autocorrelograms. Following clustering, we obtained ten distinct S1 fiber clusters, which we manually annotated on the basis of per-cluster autocorrelogram peak position (‘per-cluster’ average signals shown in Fig. 5a). These clusters classified footprinted molecules by increasing average distance between nucleosomes across entire single DNA templates, simultaneously capturing molecules with consistent NRLs (for example, NRL180–NRL357), and molecules where a regular pattern was not detected (IR1–IR4). To ascertain how cluster usage differed as a function of nucleosome density, we visualized cluster enrichment as stacked bar graphs capturing the absolute abundance of each cluster as functions of density and SNF2h or ACF remodeling (Fig. 5b).

This analysis allows us to visualize array structures on both native and remodeled fibers, to account for the random formation of nucleosome arrays by statistical positioning downstream of free DNA template ends^25^. Most prior biochemical reactions have been studied at high nucleosome densities; the products of ISWI remodeling at higher densities appear less heterogeneous than the products at lower densities. However, at these densities, the starting architecture of fibers is also less heterogeneous than at lower densities due to the effects of statistical positioning. More broadly, these results illustrate how nucleosome density can influence the state distribution of remodeling outcomes. Even at relatively low fiber densities (for example, five to seven nucleosomes per template), ISWI remodeling generates a distribution of regular fibers of various predicted NRLs, as would be predicted from a length-sensing model. Taken together, our analyses disprove the notion of ‘clamping’ for SNF2h and ACF, by (1) demonstrating that the average spacing between nucleosomes in remodeled arrays does vary significantly as a function of array density, (2) demonstrating that both remodeling reactions catalyze formation of a distribution of single-molecule array structures that are also density dependent and (3) demonstrating that these distributions are dependent on the remodeler used (that is, SNF2h versus ACF), such that ACF generates (as initially predicted by us and colleagues >16 years ago^1^) evenly spaced nucleosome arrays at lower nucleosome densities. Finally, this analysis further harmonizes our in vitro and in vivo results: ISWI remodeling in vitro generates fibers with a distribution of NRLs that scale inversely with nucleosome density, evoking the heterogeneous effects observed in mES cells.

## Discussion

### Dissecting chromatin remodeling outcomes at single-fiber resolution using SAMOSA-ChAAT

Modern chromatin biology sits amid a ‘resolution revolution’. Advances in cryogenic electron microscopy have provided us with near-atomic views of macromolecular chromatin-interacting complexes^3,45,46^. Complementarily, advances in single-molecule and high-resolution microscopic approaches in vitro and in vivo have provided new views of dynamic and often heterogeneous chromatin conformations^19,47,48^. Finally, advances in high-throughput short-read sequencing have offered near nucleotide-resolution maps of where and how these complexes engage with chromatin genome-wide, across myriad substrates in vitro, and even at the resolution of single cells^16,49–51^. SAMOSA-ChAAT provides a fourth advance in chromatin resolution—datasets describing the molecularly resolved activity of chromatin regulators on individual chromatin templates. Our data and associated computational pipelines offer a new approach for quantifying dynamic chromatin-associated processes that complement existing high-resolution approaches. We anticipate broad application of SAMOSA-ChAAT to study post-translationally modified chromatin arrays, as well as arrays undergoing additional dynamic nuclear processes (for example, transcription, replication and loop extrusion).

### ISWI remodelers sense nucleosome density

Chromatin remodelers regulate nucleosome spacing in vitro and in vivo, but the question of how chromatin remodelers space nucleosomes on individual arrays remains open. Using SAMOSA-ChAAT, we performed single-molecule-resolution footprinting experiments on reconstituted, remodeled, mammalian genomic templates of varying nucleosome density. Our in vitro results highlight two key properties of ISWI remodeling: first, remodeling outcomes are heterogeneous and largely ablate sequence-programmed nucleosome positions, consistent with prior findings that SNF2h remodeling rates are insensitive to nucleosome stability, and that remodelers can override intrinsic DNA driven nucleosome positioning^24,42^; second, ISWI remodeling products display internucleosomal distances and single-fiber nucleosome arrangements that vary as a function of underlying chromatin density. There are multiple possible explanations that could account for discrepancies between our results and prior studies demonstrating ‘clamping^17,18^.’ These include: our focus on human SNF2h and ACF (versus budding yeast), our use of single-molecule measurements that capture the structure of entire arrays (versus population averaging of MNase-digested mononucleosome positions), and our observation that at high nucleosome densities the outputs of ‘clamping’ and ‘length-sensing’ reactions appear similar, even at single-molecule resolution. As shown above, this last feature masks fundamental differences between the clamping and length-sensing models; our results further highlight the importance of single-molecule resolved measurements made at lower nucleosome densities. Finally, we note that our observation of length dependence does not preclude the possibility of ‘clamping’ having relevant regulatory effects in specific contexts (for example, different organisms) in vivo.

Our approach and results thus demonstrate the physiological relevance of the length-sensing model, by connecting DNA length-sensing on mononucleosomes to nucleosome density of individual nucleosomal arrays. At high nucleosome densities, flanking DNA is occluded and ISWI remodeling outcomes are constrained to create populations of evenly spaced arrays with short NRLs. These fiber-type distributions are probably further regulated by ISWI complex composition^29,30,52,53^. At low nucleosome densities, extranucleosomal DNA is more abundant, and nucleosome sliding by ISWI is less constrained. This can enable continuous nucleosome sliding, allowing *trans*-acting factors to overcome nucleosomal repression of regulatory DNA.

### Density-dependent remodeling explains in vivo ISWI regulatory patterns

**Fig. 6:**
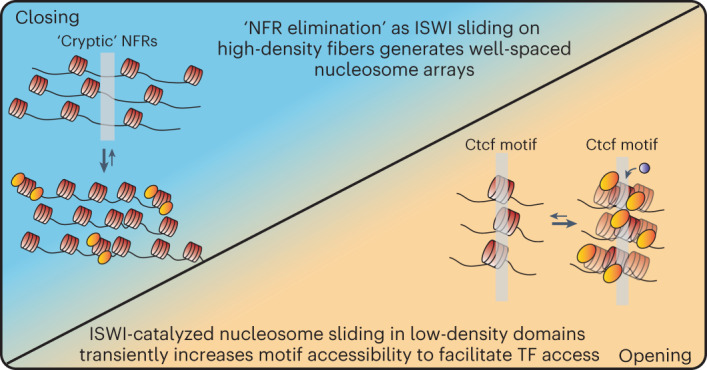
A model of SNF2h-mediated chromatin regulation based on results of this study. SNF2h length-sensing can explain context-specific regulatory functions of ISWI complexes. At high-nucleosome-density repressed regions, SNF2h-containing complexes increase the representation of multiple types of regular, short NRL fibers, presumably to facilitate elimination of cryptic NFRs. At lower-nucleosome-density regions, accessible SNF2h slides nucleosomes to increase the site exposure frequency of *cis*-regulatory elements (for example, CTCF/Ctcf binding sites).

What are the regulatory consequences of density-dependent ISWI remodeling in vivo? All of the activities discussed here, including length-dependent sliding^1,54,55^, active positioning of nucleosomes downstream of barriers^16,56^ and the formation of well-spaced nucleosome arrays^28,29^, have been noted in previous work, but how these sometimes disparate activities harmonize to impact gene regulation in vivo has remained elusive. Our data from mES cells and in vitro suggest that nucleosome density and, by extension, extranucleosomal DNA availability influence the outcomes of ISWI remodeling reactions. At regions where SNF2h maintains heterochromatic structure (that is, regions of relatively high nucleosome density), the remodeler converts irregular and long NRL fibers into well-spaced nucleosome arrays with multiple short NRLs. How well-ordered arrays repress chromatin remains unknown, but it is tempting to speculate that this process either facilitates ‘elimination’ of nucleosome-free regions (NFRs) by preventing cryptic NFR formation^57^, by promoting chromatin compaction^58^ or by generating NRLs particularly suited for phase separation^59^. At euchromatic regions where ISWI generates chromatin accessibility (that is, regions with relatively low nucleosome density), sliding can generate distributions of long NRL and ‘disordered’ arrays, to increase the site-exposure frequency of *cis-*regulatory elements such as CTCF/Ctcf binding sites (Fig. 6). Finally, our results help explain observations in both budding yeast^60^ and fruit fly^61^, of context-dependent spacing and repression by ISWI complexes. In future studies of other dynamic nuclear processes (for example, transcription, replication, repair and higher-order chromatin folding), it will be important to incorporate the role of nucleosome density in regulating ISWI outcomes.

### Nucleosome density as a long-range substrate cue for influencing chromatin remodeling activity

Our understanding of how sequence-nonspecific chromatin remodeling complexes achieve specificity at genomic loci is still developing. Prior work has uncovered myriad remodeler-targeting ‘cues’, including post-translational histone modifications^62–64^, TFs^22,50,65^, three-dimensional chromosomal architecture^66,67^ and composition of the nucleosome core particle^63,68,69^. Our work uncovers an additional cue: nucleosome density. How might nucleosome density be controlled in vivo? In mammals, nucleosome density is probably regulated at diverse length scales, ranging from local (for example, ATP-dependent chromatin remodeling; histone chaperones; histone modification; replication, transcription and repair), to global (for example, genome compartmentalization/phase separation; loop extrusion; subnuclear localization). We envision a regulatory circuit wherein the concentration of core histones can be tuned within large chromatin domains by specific *trans-* and *cis*-regulatory elements. This circuit could influence the regulatory outputs of remodeling complexes over long genomic distances, allowing higher-order genome conformation to instruct local interpretation of regulatory DNA.

## Methods

### Cloning *M. musculus* genomic sites for nucleosome array assembly

Two separate sites within the *M. musculus* reference genome containing CTCF sites were chosen for histone assembly. The CTCF genomic sites will be referred to as sequence 1 ‘S1’ (chr1:156,887,669–156,890,368, 2,712 bp) and sequence 2 ‘S2’ (chr1:156,890,410–156,893,258, 2,861 bp). S1 and S2 were polymerase chain reaction (PCR) amplified (NEBNext Q5 2× Master Mix) from purified E14 mES cell genomic DNA with primers containing homology to a Zeocin-resistance multicutter plasmid backbone as well as dual EcoRV sites for downstream separation of insert from backbone. The plasmid backbone sequence of interest containing homology was prepared with PCR amplification and the remaining parental plasmid was digested away (1 µl DpnI in 1× CutSmart at 37 °C for 1 h). All PCR products were subsequently run out on a 1% agarose gel and gel purified. After gel purification, standard Gibson Cloning for S1 or S2 inserts plus PCR-amplified/DpnI-digested backbone was performed using NEBuilder HiFi DNA Assembly Master Mix (New England Biolabs) at 3:1 insert to vector ratio. Transformation was performed with stellar competent cells (Takara) that were thawed on ice. Two microliters of assembly reaction was added to 50 µl competent cells and flicked to mix four to five times. The mixture was incubated on ice for 30 min, heat shocked at 42 °C for 30 s, and placed on ice for 2 min. A total of 950 µl SOC medium was added to the mixture, and an outgrowth step was performed at 37 °C for 1 h shaking at 1,000 RPM. The entire mixture was added to prewarmed Zeocin plates and incubated overnight. Colony PCR was performed to test for insert presence—eight colonies were selected per site and run on a 1% agarose gel. Four colonies containing the insert were selected per sequence and miniprepped overnight in low-salt Luria–Bertani broth containing zeocin (25 µg ml^−1^). Plasmids were subsequently Sanger sequenced (Genewiz) to confirm insert sequence, and one clone was selected per site for downstream experiments.

### Preparation of S1 and S2 arrays via SGD

To assemble nucleosomes onto the sequences of interest, the S1 and S2 plasmids were purified using a GigaPrep kit (Qiagen). To isolate the insert, purified plasmids were restriction enzyme digested (S1: EcoRV, ApaLI, XhoI, BsrBI and S2: EcoRV, BsrBI, BssSaI/BssSi-v2, FseI, BstXI, PflFI). Each insert was purified by size exclusion chromatography. Plasmid gigapreps were performed with a dam^+^ *Escherichia coli* strain; GATC sequences were ignored for downstream analysis of in vitro experiments. Initial restriction enzyme tests were performed with the plasmids to confirm proper digestion of the backbone, so that the insert could be purified. *Xenopus* histones were purified according to previously described methods^70^, and chromatin was assembled using SGD with varying ratios of histone:DNA.

### Purification of enzymes

The Snf2h ATPase was purified from *E. coli* and the human ACF complex was purified from Sf9 insect cells as previously described^68^. Protein concentrations were determined from SYPRO red (Thermo Fisher) staining of a sodium dodecyl sulfate (SDS)–polyacrylamide gel electrophoresis gel with bovine serum albumin standards.

### Enzyme remodeling on in vitro oligonucleosome chromatin arrays

S1 or S2 arrays assembled at varying histone:DNA concentrations (50 nM arrays) were remodeled under single-turnover, saturating enzyme conditions (9 µM SNF2h or 2 µM ACF) or under stoichiometric nucleosome:enzyme conditions (320 nM SNF2h or ACF). All remodeling reactions were performed in 12.5 mM HEPES pH 7.5, 3 mM MgCl_2_, 70 mM KCl and 0.02% NP-40. Reactions were started with the addition of saturating ATP, ADP (2 mM) or no nucleotide and incubated for 15 min at room temperature. All reactions were quenched immediately with an equal volume of ADP (34 mM) in 1× TE, resulting in 25 nM arrays.

### SAMOSA on in vitro oligonucleosome chromatin arrays

SAMOSA was performed on remodeled arrays as well as unremodeled arrays and unassembled DNA controls using the nonspecific adenine EcoGII methyltransferase (New England Biolabs, high concentration stock 2.5 × 10^4^ U ml^−1^) as previously described^9^. For the remodeled arrays, entire reaction volume was methylated with 31.25 U (1.25 µl) of EcoGII. For unremodeled arrays, 1000 nM of input was methylated with 2.5 µl EcoGII. For the unassembled, naked S1 and S2 DNA, 3 µg input DNA was methylated with 5 µl of EcoGII. Methylation reactions were performed in a 100 µl reaction containing 1× CutSmart Buffer and 1 mM *S*-adenosyl-methionine (SAM, New England Biolabs) and incubated at 37 °C for 30 min. SAM was replenished to 3.15 mM after 15 min. Unmethylated S1 and S2 naked DNA controls were similarly supplemented with Methylation Reaction buffer, minus EcoGII and replenishing SAM, and the following purification conditions. To purify the remodeled and unremodeled DNA, the samples were subsequently incubated with 10 µl Proteinase K (20 mg ml^−1^) and 10 µl 10% SDS at 65 °C for a minimum of 2 h up to overnight. To extract the DNA, equal parts volume of phenol–chloroform–isoamyl was added and mixed vigorously by shaking and then spun (maximum speed, 2 min). The aqueous portion was carefully removed, and 0.1× volume 3 M NaOAc, 3 µl of GlycoBlue and 3× volume of 100% EtOH were added, mixed gently by inversion and incubated either at −80 °C for 4 h or overnight at −20 °C. Samples were spun (maximum speed, 4 °C, 30 min), washed with 500 µl of 70% EtOH, air dried and resuspended in 50 µl EB buffer. Sample concentration was measured by Qubit High Sensitivity DNA Assay.

### Preparation of in vitro SAMOSA PacBio SMRT Libraries

The purified DNA from array and DNA samples was used in entirety as input for PacBio SMRTbell library preparation as previously described^28^. Briefly, preparation of libraries included DNA damage repair, end repair, SMRTbell ligation and Exonuclease cleanup according to the manufacturer’s instruction. After Exonuclease cleanup and a double 0.45× Ampure PB Cleanup, sample concentration was measured by Qubit High Sensitivity DNA Assay (1 µl each). To assess for library quality, samples (1 µl each) were run on the Agilent Tapestation D5000 Assay. Libraries were sequenced on Sequel II 8M SMRTcells in-house. In vitro experiment data were collected over several pooled 30 h Sequel II movie runs with either 0.6 h or 2 h pre-extension time and either 2 h or 4 h immobilization time.

### Cell lines and cell culture

Published SNF2h KO and re-expression mES cells were provided under material transfer agreement by the Dirk Schübeler Laboratory at Friedrich Miescher Institute^24^. Cells were thawed and grown for at least two passages onto CF-1 Irradiated Mouse Embryonic Feeder cells (Gibco A34181). Feeder cells were depleted from mES cells for at least two passages before collection for SAMOSA experiments. E14 mES cells were gifted from Elphege Nora Laboratory at University of California, San Francisco (UCSF). All cell lines were mycoplasma tested upon arrival, routinely tested and confirmed negative with PCR (NEBNext Q5 2× Master Mix). All feeder and mES cell cultures were grown on 0.2% gelatin. mES cells were maintained in KnockOut DMEM 1× (Gibco) supplemented with 10% fetal bovine serum (Phoenix Scientific, lot no. BW-067C18), 1% 100× GlutaMAX (Gibco), 1% 100× MEM non-essential amino acids (Gibco), 0.128 mM 2-mercaptoethanol (Bio-Rad) and 1× leukemia inhibitory factor (purified and gifted by Barbara Panning Lab at UCSF).

### SAMOSA on mES cell-derived oligonucleosomes

#### Isolation of nuclei

Nuclei were collected for the in vivo SAMOSA protocol as previously described^9^. Briefly, all nuclei were collected per cell line by centrifugation (300*g*, 5 min), washed in ice-cold 1× phosphate-buffered saline and resuspended in 1 ml Nuclear Isolation Buffer (20 mM HEPES, 10 mM KCl, 1 mM MgCl_2_, 0.1% Triton X-100, 20% glycerol and 1× protease inhibitor (Roche)) per 5–10 × 10^6^ cells by gently pipetting 5× with a wide-bore tip to release nuclei. The suspension was incubated on ice for 5 min, and nuclei were pelleted (600*g*, 4 °C, 5 min), washed with Buffer M (15 mM Tris–HCl pH 8.0, 15 mM NaCl, 60 mM KCl and 0.5 mM spermidine) and spun once again. Nuclei were counted via hemocytometer and either slow frozen or split for each experimental condition (plus or minus EcoGII methylation). To slow freeze nuclei, nuclei were resuspended in Freeze Buffer (20 mM HEPES pH 7.5, 150 mM 5 M NaCl, 0.5 mM 1 M spermidine (Sigma), 1× protease inhibitor (Roche) and 10% dimethyl sulfoxide) and stored at −80 °C.

#### Adenine methylation, MNase digest and overnight dialysis

To proceed to the modified in vivo SAMOSA protocol for direct methylation of nuclei, fresh nuclei were resuspended in Methylation Reaction Buffer (Buffer M containing 1 mM SAM). Then 200 µl methylation reactions were performed (10 µl EcoGII per 1 × 10^6^ nuclei) and incubated at 37 °C for 30 min. SAM was replenished to 6.25 mM after 15 min. Unmethylated controls were similarly supplemented with Buffer M + SAM, minus EcoGII and replenishing SAM. Samples were spun (600*g*, 4 °C, 5 min) and resuspended in cold MNase digestion Buffer (Buffer M containing 1 mM CaCl_2_). MNase digestion of nuclei was performed in 200 µl reactions, and 0.02 units of MNase was added per 1 × 10^6^ nuclei (Sigma, micrococcal nuclease from *Staphylococcus aureus*) at 4 °C for either 45 min or 1 h. Ethylene glycol tetraacetic acid was added to 2 mM to stop the digestion and incubated on ice. For nuclear lysis and liberation of chromatin fibers, MNase-digested nuclei were collected (600*g*, 4 °C, 5 min) and resuspended in ~250 µl of Tep20 Buffer (10 mM Tris–HCl pH 7.5, 0.1 mM egtazic acid, 20 mM NaCl and 1× protease inhibitor (Roche) added immediately before use) supplemented with 300 µg ml^−1^ of Lysolethicin (Sigma, l-α-lysophosphatidylcholine from bovine brain) and rotated overnight at 4 °C. Dialyzed samples were spun to remove nuclear debris (12,000*g*, 4 °C, 5 min), and soluble chromatin fibers in the supernatant were collected. Sample concentration was measured by Nanodrop, and chromatin fibers were analyzed by standard agarose gel electrophoresis.

To generate a naked DNA positive control for downstream analysis, genomic DNA was extracted from E14 mES cells with Lysis Buffer (10 mM Tris–Cl pH 8.0, 100 mM NaCl, 25 mM ethylenediaminetetraacetic acid pH 8.0, 0.5% SDS and 0.1 mg ml^−1^ Protease K) and purified with the following conditions. Methylation reactions were performed as previously stated, with 3 µg DNA as input and 5 µl EcoGII (125 U), followed by a second purification as follows. To purify all DNA samples, reactions were incubated with 10 µl of RNase A at room temperature for 10 min, followed by 10 µl Proteinase K (20 mg ml^−1^) and 10 µl 10% SDS at 65 °C for a minimum of 2 h up to overnight. To extract the DNA, equal parts volume of phenol–chloroform was added and mixed vigorously by shaking, and spun (maximum speed, 2 min). The aqueous portion was carefully removed and 0.1× volumes of 3 M NaOAc, 3 µl of GlycoBlue and 3× volumes of 100% EtOH were added, mixed gently by inversion and incubated overnight at −20 °C. Samples were then spun (maximum speed, 4 °C, 30 min), washed with 500 µl 70% EtOH, air dried and resuspended in 50 µl EB. Sample concentration was measured by Qubit High Sensitivity DNA Assay.

### Preparation of in vivo SAMOSA PacBio SMRT Libraries

Purified DNA from mES cells (methylated, unmethylated, naked DNA positive controls) was used to prepare PacBio SMRT libraries using either the SMRTbell Express Template Prep Kit 1.0 (blunt end ligation) or 2.0 (A/T overhang ligation). For the SNF2h KO and SNF2h WT AB mES cell purified SAMOSA samples, a minimum of 500 ng up to 1.5 µg was utilized as input with SMRTbell Express Template Prep Kit 1.0. For the E14 mES cells, a minimum of ~400 ng up to 1.7 µg was utilized as input with the SMRTbell Express Template Prep Kit 2.0. The naked DNA E14 positive control was sheared with a Covaris G-Tube (5424 Rotor, 3,381*g* for 1 min) and sheared to approximately 10,000 bp. Sample size distribution was checked with the Agilent Bioanalyzer DNA chip. The entire sample was utilized as input for library preparation with the PacBio SMRTbell Express Template Prep Kit 2.0. Briefly, all library preparations included DNA damage repair, end repair, SMRTbell ligation with either blunt or overhang unique adapters, and Exonuclease cleanup according to the manufacturer’s instructions. Unique PacBio SMRTbell adapters (100 µM stock) were annealed to 20 µM in annealing buffer (10 mM Tris–HCl pH 7.5 and 100 mM NaCl) in a thermocycler (95 °C 5 min, room temperature 30 min, 4 °C hold) and stored at −20 °C for long-term storage. After Exonuclease cleanup and Ampure PB cleanups (0.45× for 1.0 preparation or 1× for 2.0 preparation), the sample concentrations were measured by Qubit High Sensitivity DNA Assay (1 µl each). To assess for size distribution and library quality, samples (1 µl each) were run on an Agilent Bioanalyzer DNA chip. Libraries were sequenced in house on Sequel II 8M SMRTcells. In vivo data were collected over several pooled 30 h Sequel II movie runs with either 0.6 h or 2 h pre-extension time and either 2 h or 4 h immobilization time.

### SMRT data processing

We applied our method to two use cases in the paper, and they differ in the computational workflow to analyze them. The first is for sequencing samples where every DNA molecule has the same sequence, which is the case for our remodeling experiments on the S1 and S2 sequences. The second use case is for samples from cells containing varied sequences of DNA molecules, such as the murine in vivo experiments. The first will be referred to as homogeneous samples, and the second as genomic samples. The workflow for homogeneous samples will be presented first in each section, and the deviations for genomic samples detailed at the end.

#### Sequencing read processing

Sequencing reads were processed using software from Pacific Biosciences. The following describes the workflow for homogeneous samples:

1. Demultiplex reads
  Reads were demultiplexed using lima. The flag ‘–same’ was passed as libraries were generated with the same barcode on both ends. This produces a BAM file for the subreads of each sample.
2. Generate circular consensus sequences (CCS)
  CCS were generated for each sample using ccs. Default parameters were used other than setting the number of threads with ‘-j’. This produces a BAM file of CCS.
3. Align subreads to the reference genome
  pbmm2, the pacbio wrapper for minimap2 (ref. ^71^), was run on each subreads BAM file (the output of step 1) to align subreads to the reference sequence, producing a BAM file of aligned subreads.
4. Generate missing indices
  Our analysis code requires pacbio index files (.pbi) for each BAM file. ‘pbmm2’ does not generate index files, so missing indices were generated using ‘pbindex’.
  For genomic samples, replace step 3 with this alternate step 3.
5. Align CCS to the reference genome

Alignment was done using pbmm2, and run on each CCS file, resulting in BAM files containing the CCS and alignment information.

#### Extracting interpulse duration measurements

The interpulse duration (IPD) values were accessed from the BAM files and log_10_ transformed after setting any IPD measurements of zero frames to one frame. Then, for each zero mode waveguide, at each base in the CCS (for genomic samples) or amplicon reference (for homogeneous samples), for both strands, the log-transformed IPD values in all subreads were averaged. These mean log IPD values for the molecule were then exported along with the percentiles of log IPD values across subreads within that molecule.

### Predicting methylation status of individual adenines

#### Predicting methylation in homogeneous samples

For homogeneous samples dimensionality reduction was used to capture variation in IPD measurements between molecules, and then the reduced representations and IPD measurements were used to predict methylation. For each of S1 and S2, the non-adenine mean log IPD measurements from one unmethylated control sample were used to train a truncated singular value decomposition model. The input measurements had the mean of each base subtracted before training. The Truncated SVD class of scikit-learn was used and trained in 20 iterations to produce 40 components. The trained model was then used to transform all molecules in all samples into their reduced representations. Each resulting component had its mean subtracted and was divided by its standard deviation.

Next, a neural network model was trained to predict the mean log IPD at each base in unmethylated control molecules. The dimension reduced representation of the molecules was provided as input to the model, and the output was a value for each adenine on both strands of the amplicon molecule. The neural network was composed of four dense layers with 600 units each, with relu activation and he uniform initialization. A 50% dropout layer was placed after each of these four layers. A final dense layer produced an output for each adenine in the amplicon reference. The model was trained on a negative control sample using Keras, Adam optimizer, mean square error loss, 100 epochs and a batch size of 128. The trained model was then used to predict the mean log IPD value at all adenines in all molecules in all samples. This prediction was subtracted from the measured mean log IPD to get residuals.

A large positive residual represents slower polymerase kinetics at that adenine than would be expected given the sequence context and molecule and is thus evidence of methylation. To find a cutoff of how large the residual should be to be called as methylated, we assembled a dataset of residuals from an equal proportion of molecules from a fully methylated naked DNA control and an unmethylated control. For each individual adenine a Student’s *t*-distribution mixture model was fit to the residuals using the Python package smm. A two-component model was fit with a tolerance of 1 × 10^−6^, and a cutoff was found where that residual value was equally likely to originate from either of the two components. Adenines were then filtered by whether a sufficiently informative cutoff had been found. The three criteria for using the methylation predictions at that adenine in further analysis were as follows: (1) the mean of at least one *t*-distribution had to be above zero; (2) the difference between the means of the two *t*-distributions had to be at least *X*, where *X* was chosen separately for each amplicon reference but varied from 0.1 to 0.3; and (3) at least 2% of the training data was over the cutoff. These were lenient cutoffs that allowed the methylation predictions at ≥90% of adenines to be included in downstream analysis. This was done because the next Hidden Markov Model (HMM) step accounts for the frequency of methylation predictions in unmethylated and fully methylated control samples, and thus adenine bases where methylation prediction was poor will be less informative of DNA accessibility.

#### Predicting methylation in genomic samples

Methylation prediction was made in a similar fashion for genomic samples, with deviations necessitated by the differences in the data. Unlike in homogeneous samples, dimensionality reduction could not be used to capture intermolecular variation due to varying DNA sequences. Instead, IPD percentiles were used as neural network inputs. As described above in ‘Extracting IPD measurements’, log IPD percentiles were calculated across all subreads in each molecule separately for each template base. Every 10th percentile from 10th to 90th inclusive, for template bases C, G and T, were used as neural network input. The other input was the DNA sequence context around the measured base, given for three bases 5′ of the template adenine and ten bases 3′ of the template adenine, one-hot encoded. The neural network was a regression model predicting the measured mean log IPD at that template adenine. The neural network consisted of four dense layers with 200 units each, relu activation and he uniform initialization. The training data were 5,000,000 adenines each from six different unmethylated control samples. The validation data for early stopping were 5,000,000 adenines from each of two more unmethylated control samples. The model was trained using Keras, Adam optimizer, 20 epochs with early stopping (patience of 2 epochs) and a batch size of 128.

To determine at which adenines the methylation prediction was usefully informative and accurate, we used a second neural network model to predict the IPD residual in a positive control sample from sequence context. Sequence contexts that consistently produced residuals near zero in a positive control would be probably never methylated by EcoGII, or always methylated endogenously. The input to this network was the one-hot encoded sequence context as described above. The output was the measured log IPD with predicted log IPD subtracted. The training data were a fully methylated naked DNA sample of E14. Mean log IPD residuals were calculated using the above trained model. In total, 20,000,000 adenines were used as training data and 10,000,000 as validation data. The neural network consisted of three dense layers of 100 units, relu activation and he uniform initialization. The model was trained using Adam optimizer for two epochs with a batch size of 128. After examining the output of the trained model on negative and positive controls and chromatin, we settled on a cutoff of 0.6 for the predicted residual in positive control for calling a sequence context as usable for downstream analysis, and a cutoff of 0.42 for the mean log IPD residual for calling an adenine as methylated.

### Predicting molecule-wide DNA accessibility using HMMs

#### Predicting DNA accessibility in homogeneous samples

**Extended Data Fig. 6:**
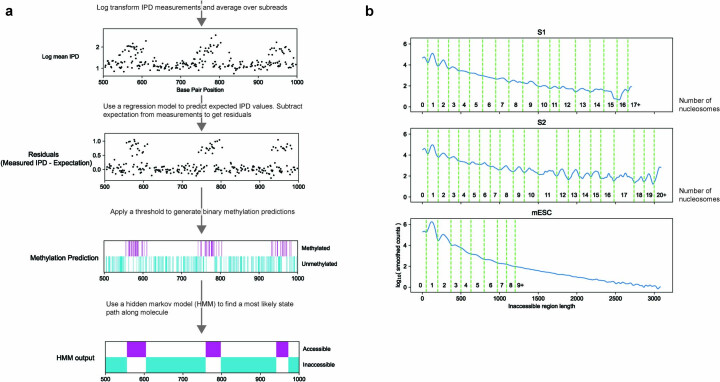
Schematics for the SAMOSA / SAMOSA-ChAAT computational pipelines. **a)**. Shown is example data for a portion of a methylated molecule containing nucleosomes assembled onto regularly spaced Widom 601 sequences. The pipeline starts with log_10_ transforming the IPD measurements and averaging over all subreads. Next, to reduce noise from DNA sequence effects and inter-molecular variation, a neural network regression model that was trained on unmethylated DNA is used to regress out the expect IPD at each adenine. The regression model takes into account the DNA sequence context as well as molecule level IPD distribution measurements. The residuals show greater signal, and a threshold is then applied to the residuals to get binary methylation predictions. A hidden Markov model (HMM) is then used to synthesize the information from all adenines across the molecule into a single trace of accessible and inaccessible regions. The HMM model uses the frequency at which adenines in different sequence contexts were methylated in unmethylated and fully methylated control molecules to set expectations for observing methylation in accessible and inaccessible regions of chromatin. This HMM output was used for all downstream analyses. **b)**. To estimate the number of nucleosomes on each DNA molecule, cutoffs were defined to delineate between the number of estimated nucleosomes within an inaccessible region. Green dashed lines show the cutoffs, and the numbers below indicate the number of nucleosomes that sized region is counted as. Different cutoffs were used for S1, S2, and mESC molecules, based on the distributions and peaks in region length for each.

To go beyond individual methylation predictions and predict DNA accessibility along each molecule we applied an HMM (Extended Data Fig. 6a). An HMM model was constructed for each amplicon reference, with two states for every adenine at which methylation was predicted: one state representing that adenine being inaccessible to the methyltransferase, and another representing it being accessible. The emission probabilities were all Bernoulli distributions, with the probability of observing a methylation in an inaccessible state being the fraction of unmethylated control molecules predicted to be methylated at that adenine, and the probability of observing a methylation in an accessible state being the fraction of fully methylated naked DNA control molecules predicted to be methylated at that adenine. To avoid any probabilities of zero, 0.5 was added to the numerator and denominator of all fractions. An initial state was created with an equal probability of transitioning into either accessible or inaccessible states. Transition probabilities between adenines were set using the logic that for an expected average duration in a single state of *L*, by the geometric distribution at each base the probability of switching states at the next base will be $\frac{1}{L}$. The probability of staying in the same state from one adenine to the next is thus ${(1-\frac{1}{L})}^{B}$, where *B* is the distance in bases between adenines. The probability of switching to the other state at the next adenine is then 1 minus that value. Different values of the average duration *L* were tested, and ultimately a value of 1,000 bp was used. This is much higher than expected, but has the beneficial result of requiring a higher burden of evidence to motivate switching states and thus minimizes spurious switching.

With the HMM model constructed, the most likely state path was found using the Viterbi algorithm for all molecules in all samples, with the predicted methylation at each adenine provided as the input. Models were constructed and solved using pomegranate^72^. The solved path was output as an array with accessible adenines as 1, inaccessible as 0, and non-adenine and uncalled bases interpolated.

#### Predicting DNA accessibility in genomic samples

In genomic samples, DNA accessibility was predicted in a similar fashion to homogeneous, except that the HMM model had to be individually constructed for each molecule due to varying DNA sequences, and rather than empirically measuring the fraction of methylation in positive and control samples at each position, neural networks were trained to predict the fraction of methylation in each from sequence context.

A neural network model was trained to predict the methylation status of adenines in the positive control sample on the basis of sequence context. The output from this model was used to approximate the probability of an adenine in that sequence context getting predicted as methylated if it was accessible to EcoGII. The sample used for training was the same naked DNA E14 methylated sample used to train the positive residual prediction model. Approximately 27,600,000 adenines were used as the training set and 7,000,000 as the validation set. The input was the one-hot encoded sequence context. The neural network consisted of three dense layers of 200 units, relu activation and he uniform initialization. The training output was binary methylation predictions, so the final output of the network had a sigmoid activation and binary cross-entropy was used as the loss. The model was trained with Adam optimizer for seven epochs with the batch size increasing each epoch from 256 to a maximum of 131,072.

An identical network was trained to predict the predicted methylation status of adenines in the unmethylated negative control samples. The output from this model was used to approximate the probability of an adenine in that sequence context getting predicted as methylated if it was not accessible to EcoGII. This one was trained using adenines combined from four different unmethylated samples, and approximately 28,100,000 adenines were used as the training set and 7,100,000 as the validation set.

The HMM models were constructed in a manner identical to that described above for homogeneous samples, except for genomic data, where an HMM model was constructed for each sequenced molecule individually. States and transition probabilities and observed output were the same. The emission probability of observing methylation at each accessible state was the output of the trained positive control methylation prediction model, and for inaccessible states was the output of the trained negative control methylation prediction model. As with homogeneous samples, the HMM was solved using the observed methylation and the Viterbi algorithm.

### Defining inaccessible regions and counting nucleosomes

**Extended Data Fig. 7:**
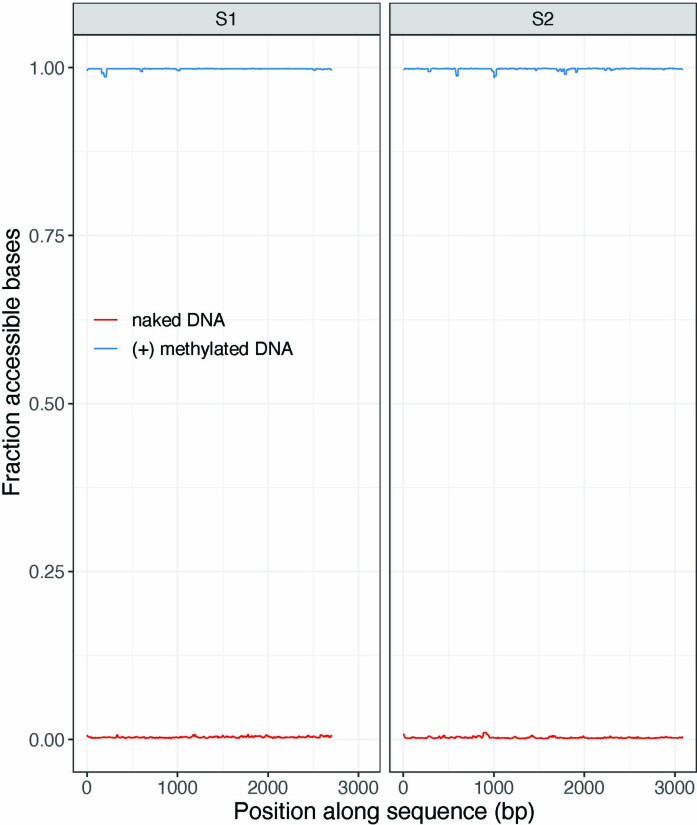
Control S1 and S2 molecules are almost entirely accessible or inaccessible based on pipeline predictions. Line plot of average accessibility of processed control S1 (left) and S2 (right) molecules, with unmethylated control DNA in red and fully methylated control DNA in blue.

Inaccessible regions were defined from the HMM output data as continuous stretches with accessibility ≤0.5. To estimate the number of nucleosomes contained within each inaccessible region, a histogram of inaccessible region lengths was generated for each data type (sequence S1, S2 and murine in vivo). Periodic peaks in these histograms were observed that approximated expected sizes for stretches containing one, two, three and so on nucleosomes. Cutoffs for the different categories were manually defined using the histogram, including a lower cutoff for subnucleosomal regions (Extended Data Fig. 6b). Importantly, the conditions we use are ‘saturating’ with calling accessibility of naked fully methylated or unmethylated molecules, as shown in Extended Data Fig. 7.

### Processed data analysis

All processed data analyses and associated scripts will be made available at GitHub^73^. Most processed data analyses proceeded from data tables generated using custom Python scripts. Resulting data tables were then used to compute all statistics reported in the paper and perform all visualizations (using tidyverse and ggplot2 in R). Below, we describe each analysis in text form, while noting that all code is freely available at the above link.

#### UMAP and Leiden clustering analyses

All UMAP and Leiden clustering analyses were performed using the scanpy package^74^. All UMAP visualizations^31^ were made using default parameters in scanpy. Leiden clustering^32^ was performed using a resolution of 0.4; clusters were then filtered on the basis of size such that all clusters that collectively summed up to <5% of the total dataset were removed. In practice, this served to remove long tails of very small clusters defined by the Leiden algorithm.

#### Signal correlation analyses

We converted footprint data files into a vector of footprint midpoint abundance for sequences S1 and S2 by summing footprint midpoint occurrences and normalizing against the total number of footprints. We then correlated these vectors across replicate experiments using R for both correlation calculations and plotting associated scatter plots.

#### Trinucleosome analyses

Using processed footprint midpoint data files, we examined, for each footprinted fiber, the distances between all consecutive footprints sized between 100 bp and 200 bp, and plotted these distances against each other. All calculations were made on processed data tables generated using scripts described in the associated Jupyter notebook.

#### Autocorrelation analyses

Autocorrelations for in vitro and in vivo data were calculated using Python, and then clustered as described above. All scripts for computing autocorrelation are available at the above link.

#### In vivo chromatin fiber analyses

All autocorrelation and clustering analyses were done as previously performed^9^. Autocorrelation and clustering were performed as above. Nucleosome density enrichment plots were generated by estimating probability distributions for background (all molecules) and cluster-specific (clustered molecules) molecules, and computing log-odds from these distributions. All per-fiber nucleosome density measurements were calculated as above. Fisher’s exact enrichment tests were carried out using scipy in Python. All *P* values calculated were then corrected using a Storey *q*-value correction, using the qvalue package in R (ref. ^75^). Multiple hypothesis correction was performed for all domain-level Fisher’s tests (including ATAC peak analyses) and cutoffs were made at *q* < 0.05.

Molecules falling within ENCODE-defined epigenomic domains were extracted using scripts published in ref. ^9^.

#### ATAC data reanalysis

SNF2hKO and AB ATAC-seq data^22^ were downloaded, remapped to mm10 using bwa, converted to sorted, deduplicated BAM files and then processed using macs2 to define accessibility peaks. Peaks were then filtered for reproducibility using the ENCODE IDR framework, and reproducible peaks were preserved for downstream analyses. Reproducible peaks for SNF2hKO and WT samples were pooled and merged using bedtools merge, and then used to generate count matrices using bedtools bamcoverage. Resulting count matrices for replicate experiments were then fed into DESeq2 to define statistically significant differentially accessible peaks with an adjusted *P-*value cutoff of 0.05.

#### Satellite sequence analyses

Detecing mouse minor (centromeric) and major (pericentromeric) satellite is challenging because of the similarity of these two sequences (including internal/self-similarity). The latter is also an issue with the telomere repeat. To use BLAST to find matches to these sequences, the output must be processed to remove overlapping matches, which is done here heuristically using an implementation of the weighted interval scheduling dynamic programming algorithm that seeks to optimize the summed bitscores for non-overlapping matches to all three sequences (minor satellite, major satellite and telomeres). This is not a perfect solution to the problem, in part because it treats the alignment for the three different repeats as effectively equivalent, and we do not believe the alignments produced by BLAST are optimal compared with, for example, Smith–Waterman alignment, and the attendant fuzziness introduced may lead to removal of a small fraction of bona fide matches.

Given the similarity of major and minor satellite sequences in particular, using the DFAM minor (SYNREP_MM, accession DF0004122.1) and major (GSAT_MM, accession DF0003028.1) satellite consensus sequences, which both exceed well-established monomer lengths of ~120 bp (minor) and ~234 bp (major), produces too many overlapping hits. Thus, we used more representative sequences from Genbank, specifically M32564.1 for major satellite and X14462.1 for minor satellite. The telomere repeat sequence was constructed by pentamerizing the telomere repeat (that is, [TTAGGG] × 5). All code used for these analyses is deposited at the above GitHub link.

#### PCA analysis

Odds ratios calculated as above were subtracted for KO and AB samples, and the resulting matrix was encoded as a numpy array object. The PCA method from scikitlearn was then used to reduce data down to four dimensions, and the first two principal components were extracted for plotting.

### Nucleosome array simulation

We implemented a Monte-Carlo simulation that iteratively places nucleosomes in random positions until a desired density is achieved, with the only constraints being that nucleosomes cannot overlap and must have at least ten nucleotides between adjacent entry/exit points. We then implemented two functions to remodel in silico: the ‘clamping’ function iterates across an array, determines whether an immediately 3′ nucleosome is ‘visible’, and then slides the 3′ nucleosome towards the 5′ nucleosome to a fixed ruler distance, which is user-defined and which is also randomly determined by sampling from a normal distribution with mean equal to the ruler distance. In this implementation, the 5′ nucleosome is always the ‘barrier’ against which a visible nucleosome is aligned. The visibility threshold used was 183 nt, and we simulated remodeling with ruler of 20 nt and ruler of 48 nt. The ‘length-sensing’ function operates on the principal that the remodeler will kinetically discriminate between flanking DNA on either side, and will only slide in a direction with sufficient flanking DNA, which is user-defined (random choice of sliding direction if there is sufficient flanking DNA on both sides). We chose minimal flanking DNA lengths of 20 nt and 48 nt based on measured biochemical rate constants for SNF2h and ACF^1^. In our implementation, each nucleosome is remodeled and nucleosomes are iteratively remodeled from 5′ to 3′. Importantly, our implementation does not capture several aspects of true ISWI remodeling—nucleosomes are remodeled in a specific order, and nucleosomes are randomly positioned without any sequence bias. Because we simulate assembly without dissociation, our Monte-Carlo simulation is subject to the packing limit as defined by Renyi^76^, which is why we simulate out to 13 nucleosomes per S1 template. Moreover, our constraint that all nucleosomes must have at least 10 bp spacing when initializing arrays specifically fails at modeling cases we know exist on S1 and S2 fibers, where two nucleosomes assemble in very close proximity to one another. Finally, we have modeled the length-sensing as a step-function gated at a particular nucleotide length; in reality, kinetic discrimination of flanking DNA would probably impact remodeling rate in a continuous fashion^1^.

### Comparing simulated and empirical per-density autocorrelograms

For scatter plots shown in Fig. 4, we specifically calculated the NRL estimate as the primary peak position for the average autocorrelation signal of single-molecules of the specified densities on the *y* axis. These are likely NRL estimates given the strength of the autocorrelation signal/peak intensities of the secondary peak. This, importantly leverages our unique ability to count individual nucleosomes on each footprinted array in SAMOSA-ChAAT data. This was specifically done to obtain as reasonable a comparison as possible across the simulated ruler, simulated length-sensing and empirical data, particularly as the simulations and empirical data alike demonstrated weak average autocorrelogram peaks at low densities. Importantly, we explore the total distributions of single-molecule autocorrelogram peaks, and the importance of clustering in interpreting these structures, in Fig. 5 and Extended Data Fig. 5. Further, we discuss how this provides even stronger support for the length-sensing model, while disproving the clamping model for SNF2h and ACF. We note that single-molecule autocorrelogram peaks should only be interpreted as NRLs / regular spacing in the context of clustering or in the context of averaged autocorrelation signal (as in Fig. 4); otherwise they are best interpreted as average distances between nucleosomes on individual fibers. This is important when interpreting similarities in mean/standard deviation in Supplementary Table 3, which manifest as substantially different cluster representations in Fig. 5, for, for example, SNF2h-remodeled arrays versus unremodeled arrays.

### Reporting summary

Further information on research design is available in the Nature Portfolio Reporting Summary linked to this article.

## Online content

Any methods, additional references, Nature Portfolio reporting summaries, source data, extended data, supplementary information, acknowledgements, peer review information; details of author contributions and competing interests; and statements of data and code availability are available at 10.1038/s41594-023-01093-6.

### Supplementary information

Reporting Summary

Supplementary Video 1 Movie of dyad-to-dyad distances for S1 SNF2h remodeling as a function of nucleosome density.

Supplementary Video 2 Movie of dyad-to-dyad distances for S12 SNF2h remodeling as a function of nucleosome density.

Supplementary Video 3 Movie of dyad-to-dyad distances for S1 ACF remodeling as a function of nucleosome density.

Supplementary Tables 1–4 Supplementary Table 1. Single-molecule peak estimates and associated standard deviation for each replicate, stratified by array type. Table 2. Summary of sequencing depths for all experiments performed in this study. For in vivo samples, ‘biorep’ refers to different biological replicate experiments, while ‘techrep’ refers to ‘technical replicates’ where the same sequencing library was sequenced on multiple PacBio Sequel II runs. Table 3. Summary of average nucleosomes and standard deviation for all *in vitro* experiments. ST refers to single-turnover remodeling reaction conditions, while MT refers to multi-turnover reaction conditions. Data are shown as mean ± standard deviation. Table 4. Mean single-molecule NRL estimates and standard deviation for all analyzed molecules in Fig. 5 and Extended Data Fig. 5.

### Source data

Source Data Fig. 4a Autocorrelations for simulated chromatin fibers after remodeling.

Source Data Extended Data Fig. 4 Source data for Extended Data Fig. 4.

## Data Availability

All processed data are available at Zenodo (10.5281/zenodo.5770727); raw data and processed data are at GEO accession GSE197979. Source data are provided with this paper.

## Code availability

All scripts and notebooks used for data analysis in this study are available at https://github.com/RamaniLab/SAMOSA-ChAAT.
