# Supplementary material for: Nucleosome density shapes kilobase-scale regulation by a mammalian chromatin remodeler
Source: Nat Struct Mol Biol. 2023 Sep 11;30(10):1571–81. doi: 10.1038/s41594-023-01093-6 (PMC10584690; doi:10.1038/s41594-023-01093-6)
Supplement: Supplementary file 1 — Reporting Summary [file 41594_2023_1093_MOESM1_ESM.pdf]

Corresponding author(s): Geeta J. Narlikar & Vijay Ramani

Last updated by author(s): Jul 4, 2023

Reporting Summary

Nature Portfolio wishes to improve the reproducibility of the work that we publish. This form provides structure for consistency and transparency in reporting. For further information on Nature Portfolio policies, see our Editorial Policies and the Editorial Policy Checklist.

Statistics

For all statistical analyses, confirm that the following items are present in the figure legend, table legend, main text, or Methods section.

n/a	Confirmed	
☐	☒	The exact sample size (*n*) for each experimental group/condition, given as a discrete number and unit of measurement
☐	☒	A statement on whether measurements were taken from distinct samples or whether the same sample was measured repeatedly
☐	☒	The statistical test(s) used AND whether they are one- or two-sided<br>*Only common tests should be described solely by name; describe more complex techniques in the Methods section.*
☒	☐	A description of all covariates tested
☒	☐	A description of any assumptions or corrections, such as tests of normality and adjustment for multiple comparisons
☐	☒	A full description of the statistical parameters including central tendency (e.g. means) or other basic estimates (e.g. regression coefficient) AND variation (e.g. standard deviation) or associated estimates of uncertainty (e.g. confidence intervals)
☐	☒	For null hypothesis testing, the test statistic (e.g. *F*, *t*, *r*) with confidence intervals, effect sizes, degrees of freedom and *P* value noted<br>*Give P values as exact values whenever suitable.*
☒	☐	For Bayesian analysis, information on the choice of priors and Markov chain Monte Carlo settings
☒	☐	For hierarchical and complex designs, identification of the appropriate level for tests and full reporting of outcomes
☐	☒	Estimates of effect sizes (e.g. Cohen's *d*, Pearson's *r*), indicating how they were calculated

Our web collection on statistics for biologists contains articles on many of the points above.

Software and code

Policy information about availability of computer code

Data collection	Commercial PacBio software (proprietary, version # not available) was used to collect basecalls for analysis.
Data analysis	All scripts & version numbers used are available on GitHub at: https://github.com/RamaniLab/SAMOSA-ChAAT

For manuscripts utilizing custom algorithms or software that are central to the research but not yet described in published literature, software must be made available to editors and reviewers. We strongly encourage code deposition in a community repository (e.g. GitHub). See the Nature Portfolio guidelines for submitting code & software for further information.

Data

Policy information about availability of data

All manuscripts must include a data availability statement. This statement should provide the following information, where applicable:

- Accession codes, unique identifiers, or web links for publicly available datasets
- A description of any restrictions on data availability
- For clinical datasets or third party data, please ensure that the statement adheres to our policy

ll processed data is available at Zenodo (https://doi.org/10.5281/zenodo.5770727); raw data and processed data is at GEO accession GSE197979.

Research involving human participants, their data, or biological material

Policy information about studies with human participants or human data. See also policy information about sex, gender (identity/presentation), and sexual orientation and race, ethnicity and racism.

Reporting on sex and gender	N/A
Reporting on race, ethnicity, or other socially relevant groupings	N/A
Population characteristics	N/A
Recruitment	N/A
Ethics oversight	N/A

Note that full information on the approval of the study protocol must also be provided in the manuscript.

Field-specific reporting

Please select the one below that is the best fit for your research. If you are not sure, read the appropriate sections before making your selection.

☒ Life sciences ☐ Behavioural & social sciences ☐ Ecological, evolutionary & environmental sciences

For a reference copy of the document with all sections, see nature.com/documents/nr-reporting-summary-flat.pdf

Life sciences study design

All studies must disclose on these points even when the disclosure is negative.

Sample size	Sample sizes were determined according to convention for in vivo experiments (i.e. two separate biological experiments per condition). Sample sizes for single-molecule experiments were not predetermined, but determined by the number of molecules sequenced on the sequencer
Data exclusions	Data were not excluded from analyses.
Replication	Replication was successful and experiments were performed at least in duplicate.
Randomization	Randomization was not used or relevant to this molecular biological / genomic study -- replicate samples were treated completely separately and used to ascertain reproducibility
Blinding	Blinding was not relevant to this study, as the data analyses performed here are all genomic analyses.

Reporting for specific materials, systems and methods

We require information from authors about some types of materials, experimental systems and methods used in many studies. Here, indicate whether each material, system or method listed is relevant to your study. If you are not sure if a list item applies to your research, read the appropriate section before selecting a response.

Materials & experimental systems

n/a	Involved in the study	
☒	☐	Antibodies
☐	☒	Eukaryotic cell lines
☒	☐	Palaeontology and archaeology
☒	☐	Animals and other organisms
☒	☐	Clinical data
☒	☐	Dual use research of concern
☒	☐	Plants

Methods

n/a	Involved in the study	
☒	☐	ChIP-seq
☒	☐	Flow cytometry
☒	☐	MRI-based neuroimaging

Eukaryotic cell lines

Policy information about cell lines and Sex and Gender in Research

Cell line source(s)	E14 cells were a gift from the Elphege Nora lab; SNF2h KO and WTAB cells were provided under MTA by the Dirk Schubeler group at FMI.
Authentication	SNF2h KO and WTAB cell lines were validated by matching molecular signal to signal presented by the Schubeler group in a prior publication. E14 cells were not authenticated.
Mycoplasma contamination	All cell lines tested negative for mycoplasma.
Commonly misidentified lines (See ICLAC register)	No cell lines used in this study are on the ICLAC register.
