# Supplementary figures and images for: Nucleosome density shapes kilobase-scale regulation by a mammalian chromatin remodeler
Source: Nat Struct Mol Biol. 2023 Sep 11;30(10):1571–81. doi: 10.1038/s41594-023-01093-6 (PMC10584690; doi:10.1038/s41594-023-01093-6)

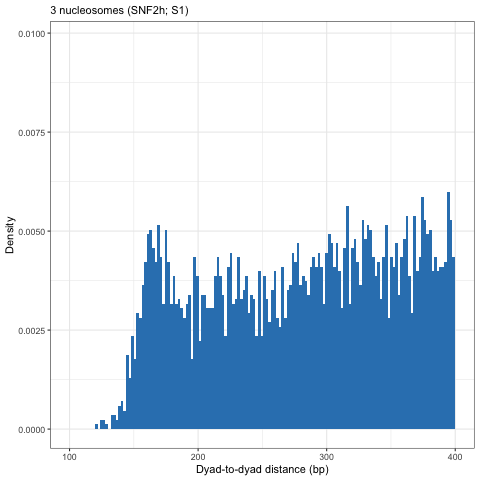

Supplement: Supplementary file 2 — Movie of dyad-to-dyad distances for S1 SNF2h remodeling as a function of nucleosome density. [file 41594_2023_1093_MOESM2_ESM.gif]

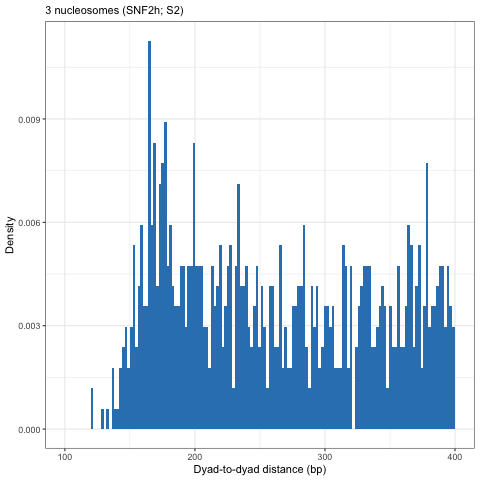

Supplement: Supplementary file 3 — Movie of dyad-to-dyad distances for S12 SNF2h remodeling as a function of nucleosome density. [file 41594_2023_1093_MOESM3_ESM.gif]

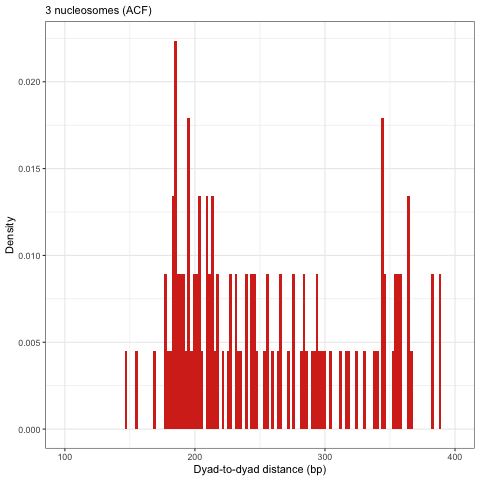

Supplement: Supplementary file 4 — Movie of dyad-to-dyad distances for S1 ACF remodeling as a function of nucleosome density. [file 41594_2023_1093_MOESM4_ESM.gif]
